# Supplementary material for: Effect of household relocation on child vaccination and health service utilisation in Dhaka, Bangladesh: a cross-sectional community survey
Source: BMJ Open. 2019 Mar 15;9(3):e026176. doi: 10.1136/bmjopen-2018-026176 (PMC6429946; doi:10.1136/bmjopen-2018-026176)
Supplement: Supplementary file 1 [file bmjopen-2018-026176supp001.pdf]

Effect of household relocation on child vaccination and health service utilization in Dhaka, Bangladesh: a cross-sectional community survey

SUPPLEMENTAL FIGURES AND TABLES

Supplemental Table 1: Household wealth principal components analysis coding and variable loading

Indicator	Coding	Loading
Number of rooms	Continuous	0.337
Housing arrangement	1 = Free<br>2 = Rental<br>3 = Owned	0.235
Roof	1 = Natural roof (bamboo/thatch)<br>2 = Rudimentary roof (tin)<br>3 = Finished roof (cement/concrete/tiled)<br>4 = Other	0.392
Walls	1 = Natural walls (jute/bamboo/mud)<br>2 = Rudimentary walls (wood)<br>3 = Finished walls (tin/brick/cement)<br>4 = Other	0.316
Floor	1 = Natural floor (earth/bamboo)<br>2 = Rudimentary floor (wood)<br>3 = Finished floor (cement/concrete)<br>4 = Other	0.377
Cooking fuel	1 = Natural (wood/grass/dung)<br>2 = Coal/charcoal<br>3 = Kerosene<br>4 = Electricity<br>5 = Gas (liquid/biogas)<br>6 = Other	0.350
Drinking water	1 = Unimproved (unprotected/surface/bottled)<br>2 = Improved but not piped<br>3 = Improved and piped into yard or private dwelling<br>4 = Other	0.170
Sanitation	1 = Unimproved (open defecation/hanging/open or broken pit)<br>2 = Improved but not piped	0.374

	3 = Improved and piped sewer<br>4 = Other	
Mobile phone ownership	0 = No<br>1 = Yes	0.380

Note: 51% of overall variance was explained by the first component.

Supplemental Table 2: ***Using vaccination card plus maternal recall*, vaccination coverage among children age 9-23 months and association with mobility status using univariate and multivariable models with modified Poisson regression**

	Partial vaccination n=518 (21%)		Full vaccination[a] n=1906 (79%)		PR[b]	95% CI	p-value
Univariate analyses							
Mobility status	n	%	n	%			
Residentially stable ≥24 months	243	19	1053	81	Reference		
Recently relocated ≤12 months	275	24	853	76	**0.93**	**0.88-0.98**	**0.009**
Demographics	mean	SE	mean	SE			
Number of children <5yrs in household	1.31	0.03	1.24	0.01	**0.94**	**0.89-0.99**	**0.035**
Age of index child in months	14.73	0.20	16.35	0.11	**1.02**	**1.02-1.03**	**<0.001**
Socioeconomics							
Mother's education	n	%	n	%			
No education	207	41	303	59	Reference		
Some schooling	121	22	433	78	**1.32**	**1.18-1.47**	**<0.001**
Finished secondary	141	16	719	84	**1.41**	**1.27-1.56**	**<0.001**
> Higher secondary	49	10	451	90	**1.52**	**1.36-1.69**	**<0.001**
Occupation of household head							
Unemployed or other	26	15	147	85	Reference		
Daily labor	195	38	318	62	**0.73**	**0.66-0.81**	**<0.001**
Shopkeeper or merchant	124	19	538	81	0.96	0.88-1.04	0.287
Salaried service	173	16	903	84	0.99	0.91-1.07	0.752
Household wealth status (PCA[c])							
Lowest	212	40	321	60	Reference		
Second	114	25	348	75	**1.25**	**1.11-1.40**	**<0.001**
Third	84	18	382	82	**1.36**	**1.20-1.54**	**<0.001**
Fourth	59	10	560	90	**1.50**	**1.34-1.69**	**<0.001**
Highest	49	14	295	86	**1.42**	**1.26-1.61**	**<0.001**
Health services knowledge							
Does *not* have knowledge of local hospital	127	28	324	72	Reference		
Has knowledge of local hospital	391	20	1582	80	**1.12**	**1.05-1.19**	**<0.001**

<u>Multivariable analyses with different models</u>				
Mobility, adjusting for demographics (# of children and age of index child)		**0.93**	**0.88-0.98**	**0.007**
Mobility, adjusting for socioeconomics (education, occupation, and wealth)		0.96	0.91-1.01	0.136
Mobility, adjusting for demographics and socioeconomics		0.96	0.92-1.01	0.138

[a]Full vaccination coverage per Expanded Programme on Immunization includes 1 dose of BCG, 3 doses of polio, 3 doses of DPT, and 1 dose of measles vaccines.
[b]PR, Prevalence Ratio.
[c]Polychoric Principal Components Analysis was used to create a household wealth index including structural housing characteristics, cooking fuel, drinking water, and sanitation.

Supplemental Table 3: ***Using vaccination card only*****, vaccination coverage among children age 9-23 months who have vaccination cards and association with mobility status using univariate and multivariable models with modified Poisson regression**

	Partial vaccination n=281 (19%)		Full vaccination[a] n=1222 (81%)		PR[b]	95% CI	p-value
Univariate analyses							
Mobility status	n	%	n	%			
Residentially stable ≥24 months	156	18	715	82	Reference		
Recently relocated ≤12 months	125	20	507	80	0.97	0.92-1.04	0.469
Demographics	mean	SE	mean	SE			
Number of children <5yrs in household	1.23	0.03	1.21	0.01	0.98	0.93-1.04	0.543
Age of index child in months	14.4	0.27	15.9	0.15	**1.02**	**1.01-1.03**	**<0.001**
Socioeconomics							
Mother's education	n	%	n	%			
No education	94	35	176	65	Reference		
Some schooling	57	17	286	83	**1.28**	**1.15-1.42**	**<0.001**
Finished secondary	93	17	461	83	**1.28**	**1.14-1.43**	**<0.001**
> Higher secondary	37	11	299	89	**1.37**	**1.22-1.52**	**<0.001**
Occupation of household head							
Unemployed or other	14	12	100	88	Reference		
Daily labor	89	33	184	67	**0.77**	**0.68-0.87**	**<0.001**
Shopkeeper or merchant	66	16	352	84	0.96	0.88-1.04	0.327
Salaried service	112	16	586	84	0.96	0.88-1.04	0.297
Household wealth status (PCA[c])							
Lowest	84	32	179	68	Reference		
Second	67	24	211	76	1.12	0.99-1.25	0.062
Third	52	17	248	83	**1.21**	**1.08-1.37**	**0.002**
Fourth	42	10	381	90	**1.32**	**1.18-1.48**	**<0.001**
Highest	36	15	203	85	**1.25**	**1.10-1.41**	**<0.001**
Health services knowledge							
Does *not* have knowledge of local hospital	55	22	194	78	Reference		
Has knowledge of local hospital	226	18	1028	82	1.05	0.98-1.13	0.141

Multivariable analyses with different models				
Mobility, adjusting for demographics (# of children and age of index child)		0.98	0.93-1.04	0.582
Mobility, adjusting for socioeconomics (education, occupation, and wealth)		1.00	0.94-1.06	0.948
Mobility, adjusting for demographics and socioeconomics		1.00	0.95-1.07	0.888

[a]Full vaccination coverage per Expanded Programme on Immunization includes 1 dose of BCG, 3 doses of polio, 3 doses of DPT, and 1 dose of measles vaccines.
[b]PR, Prevalence Ratio.
[c]Polychoric Principal Components Analysis was used to create a household wealth index including structural housing characteristics, cooking fuel, drinking water, and sanitation.
