# Supplementary figures and images for: Effect of household relocation on child vaccination and health service utilisation in Dhaka, Bangladesh: a cross-sectional community survey
Source: BMJ Open. 2019 Mar 15;9(3):e026176. doi: 10.1136/bmjopen-2018-026176 (PMC6429946; doi:10.1136/bmjopen-2018-026176)

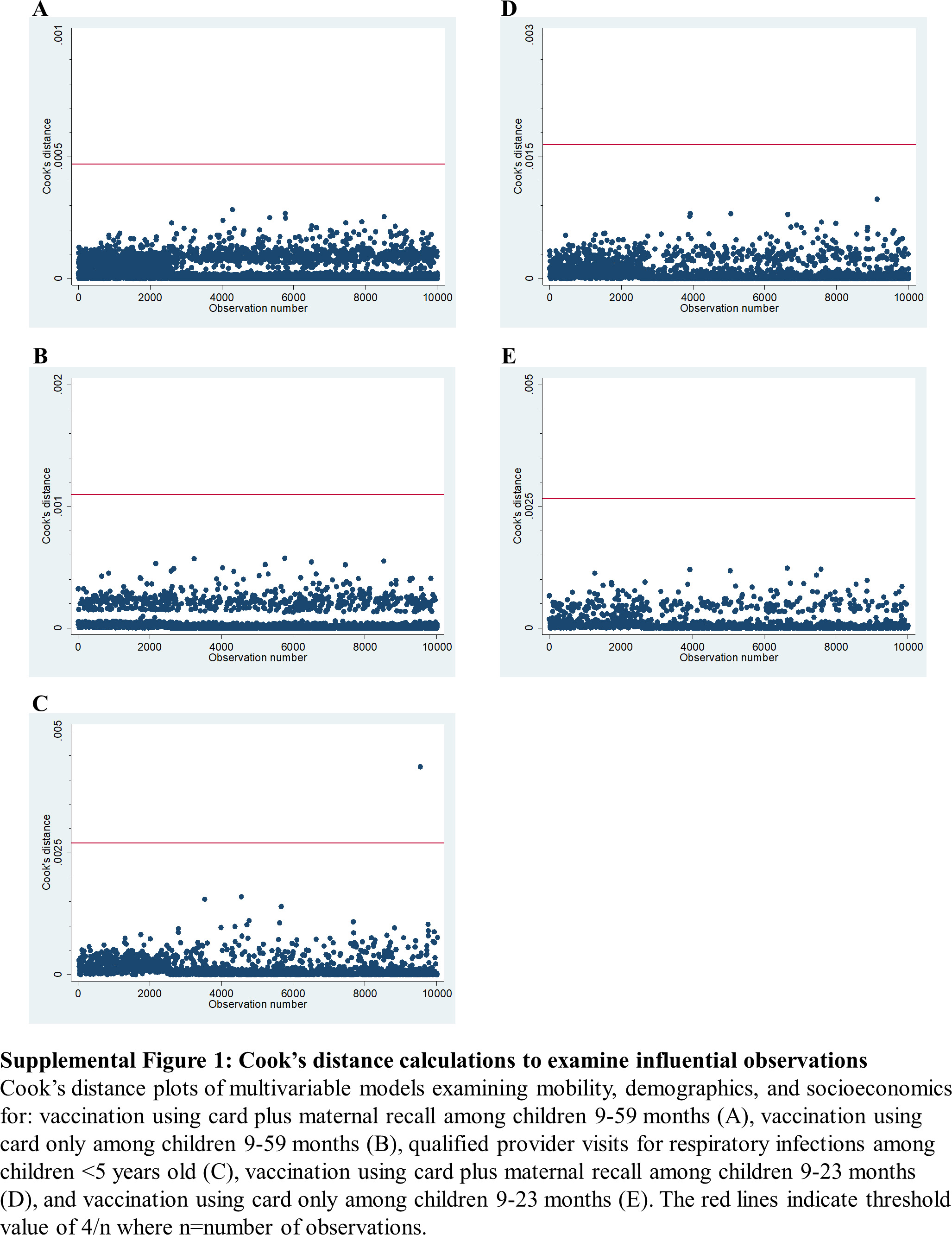

Supplement: Supplementary file 2 [file bmjopen-2018-026176supp002.jpg]
