# Supplementary material for: Effect of household relocation on child vaccination and health service utilisation in Dhaka, Bangladesh: a cross-sectional community survey
Source: BMJ Open. 2019 Mar 15;9(3):e026176. doi: 10.1136/bmjopen-2018-026176 (PMC6429946; doi:10.1136/bmjopen-2018-026176)
Supplement: Author's manuscript [file bmjopen-2018-026176.draft_revisions.pdf]

BMJ Open is committed to open peer review. As part of this commitment we make the peer review history of every article we publish publicly available.

When an article is published we post the peer reviewers' comments and the authors' responses online. We also post the versions of the paper that were used during peer review. These are the versions that the peer review comments apply to.

The versions of the paper that follow are the versions that were submitted during the peer review process. They are not the versions of record or the final published versions. They should not be cited or distributed as the published version of this manuscript.

BMJ Open is an open access journal and the full, final, typeset and author-corrected version of record of the manuscript is available on our site with no access controls, subscription charges or pay-per-view fees (http://bmjopen.bmj.com).

If you have any questions on BMJ Open's open peer review process please email info.bmjopen@bmj.com

BMJ Open

Effect of household relocation on child vaccination and health service utilization in Dhaka, Bangladesh: a cross-sectional community survey

Journal:	*BMJ Open*
Manuscript ID	bmjopen-2018-026176
Article Type:	Research
Date Submitted by the Author:	21-Aug-2018

Complete List of Authors:	Horng, Lily; Stanford University, Infectious Diseases and Geographic Medicine<br>Kakoly, Nadira Sultana; International Centre for Diarrheal Disease Research, Bangladesh (icddr, b)<br>Abedin, Jaynal ; International Centre for Diarrheal Disease Research, Bangladesh (icddr, b)<br>Luby, Stephen; Stanford University, Infectious Diseases and Geographic Medicine; International Centre for Diarrheal Disease Research, Bangladesh (icddr, b)

Keywords:	Community child health < PAEDIATRICS, PUBLIC HEALTH, Public health < INFECTIOUS DISEASES, Paediatric infectious disease & immunisation < PAEDIATRICS, EPIDEMIOLOGY

SCHOLARONE™
Manuscripts

For peer review only

Effect of household relocation on child vaccination and health service utilization in Dhaka, Bangladesh: a cross-sectional community survey

Lily M. Horng[1]*, Nadira K. Sultana[2], Jaynal Abedin[2], Stephen P. Luby[1, 2]

1 Division of Infectious Diseases & Geographic Medicine, Department of Medicine, Stanford University, Stanford, California, 94305, USA

2 International Centre for Diarrheal Disease Research, Bangladesh (icddr, b), Dhaka 1000, Bangladesh

*Corresponding author:

Lily M. Horng, MD MPH

Division of Infectious Diseases and Geographic Medicine,

Stanford University, Stanford, California, 94305, USA

+16507212730

lhorng@stanford.edu

Word count (excluding title page, abstract, references, figures and tables): 3,314 words

For peer review only

Abstract

Objective To explore the relationship between household relocation and use of vaccination and health services for severe acute respiratory illness (ARI) among children in Dhaka, Bangladesh.

Design Analysis of cross-sectional community survey data from a prior study examining the impact of *Haemophilus influenzae* type b vaccine introduction in 2009 on meningitis incidence in Bangladesh.

Setting Communities surrounding two large pediatric hospitals in Dhaka, Bangladesh.

Participants Households with children under 5 years old who either recently relocated ≤ 12 months or who were residentially stable living ≥ 24 months in their current residence (total n = 10,720) were selected for this study.

Primary outcome measures Full vaccination coverage among 9-59 month old children and visits to a qualified medical provider for severe ARI among children under 5 years old.

Results Using vaccination cards with maternal recall, full vaccination was 80% among recently relocated children (n=3,795) and 85% among residentially stable children (n=4,713) (p<0.001). Among children with ARI in the prior year, 69% of recently relocated children (n=695) had visited a qualified provider compared to 82% of residentially stable children (n=763; p<0.001). After adjusting for demographic and socioeconomic characteristics, recently relocated children were less likely to be fully vaccinated (prevalence ratio [PR] 0.97; 95% confidence interval [CI] 0.95-0.99; p=0.016) and to have visited a qualified provider for ARI (PR 0.88; 95% CI 0.84-0.93; p<0.001).

Conclusions Children in recently relocated households in Dhaka, Bangladesh have decreased use of vaccination and qualified health services for severe acute respiratory illnesses.

Strengths and limitations of this study

For peer review only - http://bmjopen.bmj.com/site/about/guidelines.xhtml

- This study examined a rich dataset from prior community surveys in Dhaka, Bangladesh to explore associations between household relocation and utilization of vaccination and qualified child health services.
- Vaccination was evaluated using different measurements and age ranges to explore trends in the relationship between mobility and vaccination.
- Effect of household relocation on use of child health services was found even after adjusting for socioeconomic factors known to impact health-seeking behavior.
- Limitations include lack of detailed data on mobility patterns and costs of health services.

For peer review only

For peer review only - http://bmjopen.bmj.com/site/about/guidelines.xhtml

Introduction

Pneumonia or acute respiratory illness (ARI) is the leading cause of death globally in children under 5 years old and caused an estimated 703,000 deaths in 2015.[1] Many causes of ARI are preventable by vaccines such as *Streptococcus pneumoniae* (attributed to 56% of global pneumonia child deaths) and *Haemophilus influenzae* type b (Hib) (8% of deaths).[1] Rapid urbanization is leading to dramatic population growth, with estimated increase of 2.6 billion people in cities by 2050 and 90% of growth projected to occur in Asia and Africa.[2] Urbanization is fueling growth of slums in which residents lack reliable access to housing, clean water, sanitation, education, and health services.[3,4] A 2006 United Nations report highlights that immunization coverage in Niger was only 35% in slums compared to 86% in non-slum urban areas.[5] In Kenya, mortality estimates from 2008-2012 for children under 5 years old were 79.8 deaths per 1,000 in Nairobi slums versus 63.4 per 1,000 in non-slum areas.[6] Multiple factors contribute to poor health in slums including contaminated environments and lack of access to appropriate services.[5,7]

Residential mobility has been recognized as an important contributor to healthcare use in high-income countries, with relocation associated with decreased use of preventive and curative services.[4,8,9] One study using a 1998 United States national health survey found that duration, distance, and frequency of moving were all predictors of decreased use of child health services even after accounting for sociodemographic factors. Households who had moved within 12 months accessed fewer preventive child health services compared to households living in their current residence over 36 months (odds ratio 3.1, 95% confidence interval [CI] 2.5-3.7).[9] Recently relocated households also accessed fewer curative services (odds ratio 3.3, 95% CI 2.6-4.2).[9] Recent migrants are often poorer, less educated, and less connected to local services.[3,4,10–13] Frequent moving also impacts children's long-term cognitive function and behavioral problems into adulthood.[14]

Few studies examine mobility and healthcare utilization in low- and middle-income countries despite high population relevance: approximately 43% of urban residents in middle-

For peer review only - http://bmjopen.bmj.com/site/about/guidelines.xhtml

income countries and 78% in low-income countries live in slums.[2,3,8,15,16] One study examining Nigeria's 2003 Demographic and Health Survey found only 9% of rural-to-urban migrant children over 12 months old were fully immunized compared to 15% of urban non-migrant children and 24% of rural non-migrant children.[16] Receipt of first dose of diphtheria, pertussis, and tetanus (DPT) vaccine recommended at 6 weeks of age was only 24% in rural-urban migrant children as compared to 47% in urban non-migrant and 63% in rural non-migrant children.[16] This association between migration and vaccination could be explained in part by socioeconomic factors as well as maternal healthcare utilization.[16]

Studying urban health services in Bangladesh is useful because Bangladesh is the world's most densely populated country that is not a city-state: the population of the capital Dhaka will increase from an estimated 16 to 27 million by 2030.[2,17] Furthermore, the government has a strong national Expanded Programme on Immunization (EPI) and active health systems research.[17,18] In 2011, full vaccination rates among children age 12-23 months were 80% nationally in Bangladesh, 75% in Dhaka, but only 43-67% in Dhaka slums.[17–21] Prior studies found that household turnover was as high as 50% in one year, comprehensive provider-led vaccination interventions were effective but too expensive to sustain, and street children were very hard to reach with interventions in Dhaka.[19,22,23]

To explore the relationship between residential mobility and healthcare utilization in Dhaka, we used data from a study showing Hib vaccine introduction into Bangladesh's EPI in 2009 dramatically reduced rates of Hib meningitis and purulent meningitis in children.[24] We conducted secondary analysis of the Hib impact study's community survey data to determine whether recently relocated children were: 1) less likely to be fully vaccinated per EPI guidelines and 2) less likely to use qualified health services for severe acute respiratory illness than residentially stable children.

Methods

For peer review only - http://bmjopen.bmj.com/site/about/guidelines.xhtml

Study design and setting

Hib conjugate vaccine was introduced into Bangladesh's EPI in 2009, and the Hib impact study conducted pre and post-vaccine surveillance of meningitis in children under 5 years old using hospital records and community surveys surrounding two large pediatric hospitals in Dhaka: Dhaka Shishu and Shishu Shastya Foundation Hospital.[24] Field researchers consecutively enrolled 100 children discharged with a diagnosis of meningitis and/or encephalitis from the two study hospitals, visited households, and recorded household geographical positional system coordinates. The catchment area was defined as the area containing >80% of households with children discharged with meningitis and within one hour of transport to either hospital. Field teams divided the catchment area into 1,748 equal-sized rectangles and randomly selected 100 rectangles as clusters. Teams surveyed each household with a child under 5 years old within 98 clusters. Two clusters were within a military cantonment, thus inaccessible. Households were asked about: 1) routine vaccinations using EPI cards and maternal recall and 2) healthcare use for children with illnesses in the prior 12 months suggestive of meningitis defined as fever plus altered mental status. Data were collected one year before (2008) and after (2010) Hib vaccine introduction.

Study population

We used the Hib impact study's pre-vaccine community surveillance data and included children based on mobility status: 1) children living in their current residence $\leq$ 12 months who we classified as "recently relocated" and 2) children living $\geq$ 24 months in their current residence who we classified as "residentially stable". We excluded children living in their current residence 13-23 months who we classified as "intermediately mobile".

Study outcomes

For peer review only - http://bmjopen.bmj.com/site/about/guidelines.xhtml

Our two primary outcomes focused on healthcare utilization: 1) full vaccination among 9-59 month old children and 2) visit to a qualified medical provider among children under 5 years old who had severe acute respiratory illness symptoms within the prior 12 months. We defined full vaccination per Bangladesh EPI guidelines in 2008 (before Hib vaccine): 1 dose of Bacillus Calmette-Guérin (BCG) vaccine against tuberculosis; 3 doses of combined vaccine against diphtheria, pertussis, and tetanus (DPT); 3 doses of oral polio vaccine (excluding polio vaccine given at birth); and 1 dose of measles vaccine. We defined severe acute respiratory illness as cough or difficulty breathing plus any danger sign: stridor, chest in-drawing, difficulty drinking/ breastfeeding, vomiting, cyanosis, convulsions, lethargy, or unconsciousness. We defined a qualified medical provider as having a Bachelor of Medicine degree or higher.

Data analysis

We compared sociodemographic and health characteristics between residentially stable and recently relocated households. For continuous variables, we calculated means with standard errors and t-tests adjusting for cluster. For categorical variables, we calculated percentages and χ^2-tests. To construct a wealth index, we used polychoric principal components analysis (PCA) including: housing (number of rooms; free, rental, or owned housing; main material of roof, walls, and floors), cooking fuel, drinking water, sanitation, and mobile phone ownership.[25–27] We then divided households into wealth quintiles. We did not include durable assets such as furniture items because ownership of these goods could be associated with duration of residency.

To examine the magnitude of association between mobility and study outcomes of vaccination and visit to a qualified provider for severe ARI, we used modified Poisson regression adjusting for cluster to estimate prevalence ratios (PRs). We conducted univariate analyses to estimate individual effects of mobility, demographics, socioeconomics, and health services knowledge (i.e. knowledge of local hospital) on healthcare utilization. Missing data

For peer review only - http://bmjopen.bmj.com/site/about/guidelines.xhtml

regarding main study outcome of acute respiratory illness were handled through listwise deletion. Given large number of missing EPI cards, we analyzed vaccination in two ways: 1) using EPI cards plus maternal recall and 2) using EPI cards alone. We conducted multivariate analyses examining the association between mobility and healthcare utilization, adjusting for demographics and socioeconomics known to influence health-seeking behavior.[7,10,11,13]

Ethics

The Ethical Review Committee of the International Centre for Diarrhoeal Disease Research, Bangladesh (icddr,b) reviewed and approved the study protocol. Written informed consent was obtained from all participants before taking part in the initial Hib impact study.

Patient and public involvement

No participants were directly involved in development of the research questions and outcomes. No participants were involved in the design or conduct of the study. There are no plans to disseminate the results of the research individually to study participants.

Results

We surveyed a total of 10,720 households with children less than 5 years old: 42% of households had recently relocated within 12 months, 51% were residentially stable living in their current residence over 24 months, and 7% were intermediately mobile (Figure 1). We excluded the 700 intermediately mobile children from subsequent analyses. For the healthcare utilization analysis, 1,458 children had severe ARI symptoms within the 12 months prior to survey. For the vaccination analysis, 8,508 children were age 9-59 months and thus should have completed all EPI-recommended vaccinations.

Recently relocated families had smaller households, less education, less wealth, and less knowledge of the local hospital compared to residentially stable families (Table 1).

For peer review only - http://bmjopen.bmj.com/site/about/guidelines.xhtml

Recently relocated families were poorer: 24% earned less than 5,000 Bangladeshi taka (US $73) per month compared to 18% of residentially stable families. Household income was missing for 12 out of 10,020 households. For the wealth index analysis, the first principal component accounted for 51% of overall variance, with largest contributions from roof and floor materials, sanitation, and mobile phone ownership (Supplemental Table 1). Among recently relocated families, 48% were in the two poorest wealth quintiles compared to 37% of residentially stable families. Fewer recently relocated caregivers had knowledge of the local hospital, 76%, compared to residentially stable caregivers, 85%. Similar rates of illness in the 12 months prior to survey were reported by all households: 14-15% of children with symptoms of severe ARI and 3-4% with symptoms of meningitis/encephalitis.

Full vaccination coverage measured by EPI card plus maternal recall was 83% among all children age 9-59 months (Table 2). Full vaccination was 80% among recently relocated children and 85% among residentially stable children (univariate PR 0.94, 95% CI 0.91-0.97, $p<0.001$). Vaccination was lower in households with more children and younger children. Socioeconomic factors, especially mother's education, had the strongest association with vaccination. In multivariate analyses, recently relocated children were 3% less likely than residentially stable children to be fully vaccinated even after adjusting for demographic and socioeconomic factors (multivariate PR 0.97, 95% CI 0.95-0.99, $p=0.016$).

Vaccination was also analyzed using only EPI cards (Table 3). At time of survey, only 43% of all children had EPI cards available. Fewer recently relocated children had EPI cards, 36%, compared to 48% of residentially stable children ($p<0.001$). Full vaccination per EPI card was 83% among recently relocated children and 86% among residentially stable children ($p=0.083$). The 9-59 month age range for full vaccination analysis allowed inclusion of a larger sample size of children vulnerable to vaccine-preventable diseases. Narrowing the age range to 9-23 months showed similar results although with smaller sample sizes limiting statistical power to detect mobility-vaccination associations (Supplementary Tables 2 and 3).

For peer review only - http://bmjopen.bmj.com/site/about/guidelines.xhtml

In addition, using a 10-month age minimum to account for potential delay in measles vaccination showed the same results as a 9-month age cutoff (data not shown).

Among all children under 5 years old with severe ARI in the past year, 75% visited a qualified medical provider (Table 4). Fewer recently relocated children with severe ARI saw a qualified provider, 69%, as compared to 82% of residentially stable children (univariate PR 0.84, 95% CI 0.79-0.90, p<0.001). Socioeconomic factors, especially household wealth, were strongly associated with qualified provider visits.

Health services knowledge was also strongly associated with acute healthcare visits: 80% of parents who knew about the local hospital sought ARI treatment from a qualified provider as compared to 51% of parents who did *not* have knowledge of the local hospital (univariate PR 1.57, 95% CI 1.34-1.85, p<0.001). After adjusting for demographic and socioeconomic factors, recently relocated households were 11% less likely than residentially stable households to visit a qualified medical provider for children with severe ARI (multivariate PR 0.88, 95% CI 0.84-0.93, p<0.001).

Discussion

Recently relocated households were less likely to use both acute and preventive child healthcare services in our study in Dhaka, Bangladesh, and these findings support prior literature exploring the effects of mobility on healthcare utilization.[8,9,14,16] Household relocation had a strong association with decreased use of qualified medical services for severe acute respiratory illness. Similarly, household relocation was associated with decreased vaccination rates although this relationship was less robust. Another key finding was that recently relocated parents were less knowledgeable about the local hospital compared to residentially stable parents, and knowledge of the local hospital had as strong an association with acute healthcare visits as some economic factors. Overall, recently relocated children in our study had slightly lower vaccination rates and markedly lower use of acute healthcare services for ARI than residentially stable children.

For peer review only - http://bmjopen.bmj.com/site/about/guidelines.xhtml

Study strengths include data focused on urban Bangladesh and exploring vaccination status using different measurements as well as adjusting for socioeconomic factors when examining mobility and health service utilization. Our study used the Hib impact study's rigorous community surveillance data of households living close to tertiary care pediatric hospitals in Dhaka. Dhaka residents have high mobility and many options for healthcare. Unlike in rural areas, physical access to health services is usually *not* a barrier to healthcare use in urban areas. One study found almost all residents in Dhaka lived within 1 kilometer of primary health services.[28] Routine immunizations are provided free by the government of Bangladesh, but acute care services require out of pocket expenditures which can be a barrier to access. Our findings on mobility and child health services use in Dhaka could inform health services work in other urban low- and middle-income country contexts.[2,6,7]

We analyzed vaccination using EPI card data augmented with maternal recall, EPI card data only, and several different age ranges. Vaccination status can be difficult to measure in community surveys. Written documentation of vaccination is objective and easy to measure, but EPI card retention can be affected by parental education, household wealth, age of child, and even household relocation. Although maternal recall can be influenced by education, social desirability bias, and vaccine-specific knowledge, studies in low- and middle-income countries show high correlation between maternal recall and EPI cards.[29–31] Using only EPI cards or narrower age ranges in our vaccination analyses resulted in smaller sample sizes which limited statistical power, but all analyses showed similar effect estimates of increased mobility associated with decreased vaccination. Moreover, the association between increased household relocation and decreased health services use was still seen even after adjusting for socioeconomic factors known to impact healthcare use.

Study limitations include lack of data on mobility patterns and health services costs. Information on households' prior residences, distances moved, or frequency of moving was not available in our dataset. Households moving from rural Bangladesh to urban Dhaka, moving long distances, or relocating frequently probably have less knowledge and therefore

For peer review only - http://bmjopen.bmj.com/site/about/guidelines.xhtml

use of locally available health services.[9] Our findings thus likely underestimate the association between mobility and healthcare utilization for households with large migration changes. We were also unable to examine household relocation timing in relation to healthcare use. Recently relocated households were not asked if healthcare visits occurred before or after moving. Healthcare visits before moving would not be relevant to how mobility affects use of health services after moving. Our findings likely underestimate the association between mobility and health-seeking behavior. Ultimately, our results show a modest overall association between mobility and healthcare use which could be elucidated by asking about migration patterns including timing of use of health services.

Our dataset did not contain cost of services, which is a well-known barrier to healthcare use.[11,20,21] Although vaccinations are provided for free, some non-governmental and private organizations charge fees for patient registration. Even small fees could have lowered vaccination rates. Cost of services, willingness to pay, and underlying finances are strongly linked, thus adjusting for socioeconomic factors of parental education and wealth in our models should have incorporated some cost effects on healthcare use. However, costs could affect recently relocated households disproportionately more than residentially stable households of the same socioeconomic status. One could hypothesize that immediately after relocating, families would first spend money on household goods before preventive medicine fees. Without cost data, we can still conclude from our analysis that increased mobility is associated with decreased healthcare use, but we have limited understanding of mechanisms through which mobility affects healthcare use.

Barriers and delays to using appropriate healthcare services increase mortality.[11,32] One study in India of 290 children hospitalized for pneumonia in a tertiary care center found that delayed hospital referral, defined as three or more days between symptom onset and hospitalization, was associated with increased mortality (OR 52.1, 95% CI 6.7-402.4, $p<0.001$) after adjusting for age, residence in slum, and illness severity.[32] In this study, incomplete immunization was also associated with increased mortality (OR 12.3, 95% CI 2.2-

For peer review only - http://bmjopen.bmj.com/site/about/guidelines.xhtml

69.9, p=0.005).[32] Reasons for delayed care-seeking can include access and cost. While cost does influence healthcare use, parents of sick children usually do seek some treatment. In the 2014 Bangladesh Demographic and Health Survey, 52% of urban parents with children with ARI symptoms in the 2 weeks prior to survey sought treatment from a health facility, 23% went to pharmacies, and 12% went to traditional practitioners.[33] Only 12% of parents with sick children sought no health care treatment at all.[33] While cost does not seem a large barrier to seeking any treatment at all, cost likely influences choice of health provider.

Household relocation disrupts prior relationships with healthcare providers and results in lack of familiarity with local services. Studies show that continuity of care is associated with increased vaccination, fewer emergency department visits, and decreased hospitalization among children.[9,34–36] People usually move to new areas because of pre-existing social connections through family, friends, or work.[4,37] These social contacts can act as pathways of important local knowledge, including health services, but recently relocated households have fewer social contacts and access fewer information sources. Other studies have also found that parental attitudes and knowledge are critical factors contributing to use of health services.[38–40] One literature review found that *practical* knowledge about vaccination schedule, timing, and logistics had a stronger association with vaccination uptake than scientific knowledge of vaccine names or biologic actions.[40]

Our finding that recently relocated children in Dhaka use fewer qualified health services compared to residentially stable children sheds light on health barriers faced by a growing population of children living in urban centers of low- and middle-income countries. Policymakers working to improve urban child health could invest in accurate counting of children living in communities with high household turnover in order to connect recently relocated households to already existing local health services. Further studies by researchers on patterns and mechanisms through which mobility affects healthcare use could inform critical intervention points. Ultimately, cost-effective and targeted interventions to increase

For peer review only - http://bmjopen.bmj.com/site/about/guidelines.xhtml

appropriate healthcare use among recently relocated children could improve health of future urban populations.

Contributors

SL was the Principal Investigator and involved in every aspect of the study from conceptualization to data analysis to manuscript writing and submission. NS, JA, and LH were involved in data analysis, and LH drafted the written work. All authors collaborated on and approved the final manuscript.

Competing interests

None declared.

Provenance and peer review

Not commissioned; externally peer reviewed.

Ethical considerations

The Ethical Review Committee of the International Centre for Diarrhoeal Disease Research, Bangladesh (icddr,b) reviewed and approved the study protocol.

Participant consent

Written informed consent was obtained from all participants before taking part in the initial Hib impact study.

Funding

The original Hib impact study data collection was funded by the Bill and Melinda Gates Foundation and the GAVI Hib Initiative (GR-00580). No additional funding was provided for this study.

For peer review only

For peer review only - http://bmjopen.bmj.com/site/about/guidelines.xhtml

Data sharing

Data are available by emailing the corresponding author LH at lhorng@stanford.edu.

Acknowledgements

The authors thank study participants, field researchers, and collaborative partners of the Hib impact study who included Dhaka Shishu microbiology laboratories and the Johns Hopkins Bloomberg School of Public Health. icddr,b is grateful to the Bangladesh, Canada, Sweden and the United Kingdom governments for providing core/unrestricted support.

For peer review only

For peer review only - http://bmjopen.bmj.com/site/about/guidelines.xhtml

References

1 Wang H, Naghavi M, Allen C, *et al.* Global, regional, and national life expectancy, all-cause mortality, and cause-specific mortality for 249 causes of death, 1980–2015: a systematic analysis for the Global Burden of Disease Study 2015. *Lancet* 2016;**388**:1459–544.

2 United Nations, Department of Economic and Social Affairs, Population Division. World Urbanization Prospects: The 2014 Revision. New York: 2014. http://esa.un.org/unpd/wup/Publications/Files/WUP2014-Report.pdf (accessed 3 Feb 2016).

3 United Nations Human Settlements Programme. The challenge of slums: global report on human settlements 2003. *UN-HABITAT* 2003.

4 Rashid SF. Strategies to reduce exclusion among populations living in urban slum settlements in Bangladesh. *J Health Popul Nutr* 2009;**27**:574–86.

5 Habitat UN. State of the World's Cities 2006/7. *New York: United Nations* 2006.

6 African Population and Health Research Center (APHRC). Population and health dynamics in Nairobi's informal settlements: report of the Nairobi Cross-sectional Slums Survey (NCSS) 2012. Nairobi: : African Population and Health Research Center (APHRC) 2014.

7 Unger A. Children's health in slum settings. *Arch Dis Child* 2013;**98**:799–805. doi:10.1136/archdischild-2011-301621

8 Jelleyman T, Spencer N. Residential mobility in childhood and health outcomes: a systematic review. *J Epidemiol Community Health* 2008;**62**:584–92.

9 Fowler MG, Simpson GA, Schoendorf KC. Families on the move and children's health care. *Pediatrics* 1993;**91**:934–40.

10 Quaiyum MA, Gazi R, Khan AI, *et al.* Programmatic aspects of dropouts in child vaccination in Bangladesh: findings from a prospective study. *Asia Pac J Public Health* 2011;**23**:141–50. doi:10.1177/1010539509342119

11 Hussain A, Ali SM, Kvåle G. Determinants of mortality among children in the urban slums of Dhaka city, Bangladesh. *Trop Med Int Health* 1999;**4**:758–64.

12 Awoh AB, Plugge E. Immunisation coverage in rural–urban migrant children in low and middle-income countries (LMICs): a systematic review and meta-analysis. *J Epidemiol Community Health* 2016;**70**:305–11.

13 Kusuma YS, Kumari R, Pandav CS, *et al.* Migration and immunization: determinants of childhood immunization uptake among socioeconomically disadvantaged migrants in Delhi, India. *Trop Med Int Health* 2010;**15**:1326–32.

14 Wood D, Halfon N, Scarlata D, *et al.* Impact of family relocation on children's growth, development, school function, and behavior. *JAMA* 1993;**270**:1334–8.

15 Patel RB, Burke TF. Urbanization--an emerging humanitarian disaster. *N Engl J Med* 2009;**361**:741–3. doi:10.1056/NEJMp0810878

For peer review only

For peer review only - http://bmjopen.bmj.com/site/about/guidelines.xhtml

16 Antai D. Migration and child immunization in Nigeria: individual-and community-level contexts. *BMC Public Health* 2010;**10**:116.

17 NIPORT, icddr,b, MEASURE Evaluation. Bangladesh Urban Health Survey 2013 Final Report. 2015. http://www.cpc.unc.edu/measure/resources/publications/tr-15-117 (accessed 3 Feb 2016).

18 Government of Bangladesh. Bangladesh EPI coverage evaluation survey 2011, Expanded Programme on Immunization. 2011. http://www.dghs.gov.bd/images/docs/EPI/CES%20Reports/EPI%20Coverage%20Evaluation%20Survey%202011.pdf

19 Hayford K, Uddin MJ, Koehlmoos TP, *et al.* Cost and sustainability of a successful package of interventions to improve vaccination coverage for children in urban slums of Bangladesh. *Vaccine* 2014;**32**:2294–9. doi:10.1016/j.vaccine.2014.02.075

20 Uddin MJ, Larson CP, Oliveras E, *et al.* Child immunization coverage in urban slums of Bangladesh: impact of an intervention package. *Health Policy Plan* 2010;**25**. doi:10.1093/heapol/czp041

21 Perry H, Weierbach R, Hossain I, *et al.* Childhood immunization coverage in zone 3 of Dhaka City: the challenge of reaching impoverished households in urban Bangladesh. *Bull World Health Organ* 1998;**76**:565–73.

22 Luby SP, Halder AK, Saha SK, *et al.* A low-cost approach to measure the burden of vaccine preventable diseases in urban areas. *Vaccine* 2010;**28**:4903–12. doi:10.1016/j.vaccine.2010.05.040

23 Uddin MJ, Shamsuzzaman M, Horng L, *et al.* Use of mobile phones for improving vaccination coverage among children living in rural hard-to-reach areas and urban streets of Bangladesh. *Vaccine* 2016;**34**:276–83. doi:10.1016/j.vaccine.2015.11.024

24 Sultana NK, Saha SK, Al-Emran HM, *et al.* Impact of introduction of the Haemophilus influenzae type b conjugate vaccine into childhood immunization on meningitis in Bangladeshi infants. *J Pediatr* 2013;**163**:S73–8.

25 Vyas S, Kumaranayake L. Constructing socio-economic status indices: how to use principal components analysis. *Health Policy Plan* 2006;**21**:459–68. doi:10.1093/heapol/czl029

26 Luby SP, Halder AK. Associations among handwashing indicators, wealth, and symptoms of childhood respiratory illness in urban Bangladesh. *Trop Med Int Health* 2008;**13**:835–44. doi:10.1111/j.1365-3156.2008.02074.x

27 Sharker MY, Nasser M, Abedin J, *et al.* The risk of misclassifying subjects within principal component based asset index. *Emerg Themes Epidemiol* 2014;**11**:6.

28 Heller LR. Do slums matter? Location and early childhood preventive care choices among urban residents of Bangladesh. *Soc Sci Med* 2013;**94**. doi:10.1016/j.socscimed.2013.06.011

29 Murray CJ, Shengelia B, Gupta N, *et al.* Validity of reported vaccination coverage in 45 countries. *Lancet* 2003;**362**:1022–7.

For peer review only - http://bmjopen.bmj.com/site/about/guidelines.xhtml

30 Miles M, Ryman TK, Dietz V, *et al.* Validity of vaccination cards and parental recall to estimate vaccination coverage: a systematic review of the literature. *Vaccine* 2013;**31**:1560–8. doi:10.1016/j.vaccine.2012.10.089

31 Brown J, Monasch R, Bicego G, *et al. An assessment of the quality of national child immunization coverage estimates in population-based surveys*. MEASURE Evaluation, Carolina Population Center, University of North Carolina at Chapel Hill 2002.

32 Bokade CM, Madhura AD, Bagul AS, *et al.* Predictors of mortality in children due to severe and very severe pneumonia. *Niger Med J* 2015;**56**:287.

33 National Institute of Population Research and Training - NIPORT/Bangladesh, Mitra and Associates, ICF International. *Bangladesh Demographic and Health Survey 2014*. Dhaka, Bangladesh: : NIPORT, Mitra and Associates, and ICF International 2016. http://dhsprogram.com/pubs/pdf/FR311/FR311.pdf

34 Christakis DA, Mell L, Wright JA, *et al.* The association between greater continuity of care and timely measles-mumps-rubella vaccination. *Am J Public Health* 2000;**90**:962.

35 Christakis DA, Mell L, Koepsell TD, *et al.* Association of lower continuity of care with greater risk of emergency department use and hospitalization in children. *Pediatrics* 2001;**107**:524–9.

36 Van Walraven C, Oake N, Jennings A, *et al.* The association between continuity of care and outcomes: a systematic and critical review. *J Eval Clin Pract* 2010;**16**:947–56.

37 Kuhn R. Identities in motion: Social exchange networks and rural-urban migration in Bangladesh. *Contrib Indian Sociol* 2003;**37**:311–37.

38 Mills E, Jadad AR, Ross C, *et al.* Systematic review of qualitative studies exploring parental beliefs and attitudes toward childhood vaccination identifies common barriers to vaccination. *J Clin Epidemiol* 11;**58**:1081–8. doi:10.1016/j.jclinepi.2005.09.002

39 Rainey JJ, Watkins M, Ryman TK, *et al.* Reasons related to non-vaccination and under-vaccination of children in low and middle income countries: findings from a systematic review of the published literature, 1999–2009. *Vaccine* 2011;**29**:8215–21.

40 Favin M, Steinglass R, Fields R, *et al.* Why children are not vaccinated: a review of the grey literature. *Int Health* 2012;**4**:229–38.

For peer review only

For peer review only - http://bmjopen.bmj.com/site/about/guidelines.xhtml

Tables

Table 1: **Mobility status of study households with children age 0-59 months and association with demographic, socioeconomic, and health characteristics using t- and χ^2-tests**

	Residentially stable, ≥24 months n=5513		Recently relocated, ≤12 months n=4507		p-value
Demographics	mean	SE	mean	SE	
Number of household members	5.40	0.09	4.60	0.10	**<0.001**
Number of children <5yrs in household	1.28	0.01	1.25	0.01	0.194
Age of index child in months	30.0	0.23	28.7	0.25	**<0.001**
	n	%	n	%	
Sex of index child: Male	2478	45	2048	45	0.622
Socioeconomics					
Mother's education					**<0.001**
No education	1133	21	1162	26	
Some schooling	1142	21	1176	26	
Finished secondary	1849	34	1483	33	
> Secondary	1389	25	686	15	
Father's education					**<0.001**
No education	1261	23	1139	25	
Some schooling	913	17	911	20	
Finished secondary	1525	28	1372	30	
> Secondary	1814	33	1085	24	
Occupation of household head					**<0.001**
Unemployed or other	482	9	232	5	
Daily labor	916	17	1218	27	
Shopkeeper or merchant	1787	32	1058	23	
Salaried service	2328	42	1999	44	
Monthly household income[a]					**<0.001**
≤ 5,000 taka (US $73)	971	18	1080	24	

For peer review only

For peer review only - http://bmjopen.bmj.com/site/about/guidelines.xhtml

5,001 – 10,000 taka	1634	30	1925	43	
> 10,000 taka (US $145)	2900	53	1498	33	
Household wealth index[b]					**<0.001**
Poorest	1092	20	1110	25	
Lower middle	922	17	1021	23	
Middle	966f	18	911	20	
Upper middle	1455	26	1155	26	
Richest	1078	20	310	7	
Health services knowledge					
Knowledge of local hospital	4709	85	3428	76	**<0.001**
Health outcomes					
Severe acute respiratory illness suffered by index child within 12 months[c]	763	14	695	15	**0.026**
Meningitis/encephalitis suffered by index child within 12 months[d]	185	3	164	4	0.443

[a]12 respondents (8 residentially stable and 4 recently relocated) did not know or did not disclose household income.
[b]Polychoric Principal Components Analysis was used to create a household wealth index including structural housing characteristics, cooking fuel, drinking water, and sanitation.
[c]One residentially stable respondent did not know if child recently had a severe acute respiratory illness.
[d]Three respondents did not know if child recently had a serious illness with mental status changes (2 residentially stable and 1 recently relocated).

For peer review only

Table 2: *Using EPI card plus maternal recall*, vaccination coverage among children age 9-59 months and association with mobility status using univariate and multivariate models with modified Poisson regression

	Partial vaccination n=1465 (17%)		Full vaccination[a] n=7043 (83%)		PR[b]	95% CI	p-value
Univariate analyses							
Mobility status	n	%	n	%			
Residentially stable ≥24 months	706	15	4007	85	Reference		
Recently relocated ≤12 months	759	20	3036	80	**0.94**	**0.91-0.97**	**<0.001**
Demographics	mean	SE	mean	SE			
Number of children <5yrs in household	1.31	0.02	1.25	0.01	**0.96**	**0.93-0.98**	**0.002**
Age of index child in months	32.0	0.44	34.2	0.16	**1.002**	**1.001-1.003**	**<0.001**
Socioeconomics							
Mother's education	n	%	n	%			
No education	629	31	1377	69	Reference		
Some schooling	401	21	1540	79	**1.16**	**1.10-1.21**	**<0.001**
Finished secondary	323	11	2513	89	**1.29**	**1.23-1.36**	**<0.001**
> Higher secondary	112	6	1613	94	**1.36**	**1.30-1.43**	**<0.001**
Occupation of household head							
Unemployed or other	106	17	505	83	Reference		
Daily labor	524	29	1283	71	**0.86**	**0.82-0.90**	**<0.001**
Shopkeeper or merchant	348	14	2084	86	1.04	0.99-1.08	0.112
Salaried service	487	13	3171	87	**1.05**	**1.01-1.09**	**0.029**
Household wealth status (PCA[c])							
Poorest	596	32	1290	68	Reference		
Lower middle	334	20	1298	80	**1.16**	**1.09-1.24**	**<0.001**
Middle	242	15	1348	85	**1.24**	**1.16-1.32**	**<0.001**
Upper middle	193	9	2043	91	**1.34**	**1.25-1.42**	**<0.001**
Richest	100	9	1064	91	**1.34**	**1.25-1.43**	**<0.001**
Health services knowledge							
Does *not* have knowledge of local hospital	354	23	1218	77	Reference		
Has knowledge of local hospital	1111	16	5825	84	**1.08**	**1.05-1.12**	**<0.001**

For peer review only

For peer review only - http://bmjopen.bmj.com/site/about/guidelines.xhtml

Multivariate analyses with different models			
Mobility, adjusting for demographics (# of children and age of index child)	**0.94**	**0.92-0.97**	**<0.001**
Mobility, adjusting for socioeconomics (education, occupation, and wealth)	**0.97**	**0.95-0.99**	**0.009**
Mobility, adjusting for demographics and socioeconomics	**0.97**	**0.95-0.99**	**0.016**

[a]Full vaccination coverage per EPI program includes 1 dose of BCG, 3 doses of polio, 3 doses of DPT, and 1 dose of measles vaccines.
[b]PR, Prevalence Ratio.
[c]Polychoric Principal Components Analysis was used to create a household wealth index including structural housing characteristics, cooking fuel, drinking water, and sanitation.

For peer review only

For peer review only - http://bmjopen.bmj.com/site/about/guidelines.xhtml

Table 3: *Using EPI card only*, vaccination coverage among children age 9-59 months who have EPI cards and association with mobility status using univariate and multivariate models with modified Poisson regression

	Partial vaccination n=564 (15%)		Full vaccination[a] n=3085 (85%)		PR[b]	95% CI	p-value
Univariate analyses							
Mobility status	n	%	n	%			
Residentially stable ≥24 months	329	14	1948	86	Reference		
Recently relocated ≤12 months	235	17	1137	83	0.97	0.93-1.00	0.083
Demographics	mean	SE	mean	SE			
Number of children <5yrs in household	1.26	0.02	1.24	0.01	0.99	0.96-1.02	0.486
Age of index child in months	27.1	0.64	29.6	0.25	**1.002**	**1.001-1.003**	**0.001**
Socioeconomics							
Mother's education	n	%	n	%			
No education	165	28	430	72	Reference		
Some schooling	123	16	654	84	**1.16**	**1.09-1.24**	**<0.001**
Finished secondary	186	14	1158	86	**1.19**	**1.11-1.28**	**<0.001**
> Higher secondary	90	10	843	90	**1.25**	**1.17-1.33**	**<0.001**
Occupation of household head							
Unemployed or other	35	13	238	87	Reference		
Daily labor	148	26	413	74	**0.84**	**0.78-0.91**	**<0.001**
Shopkeeper or merchant	143	13	921	87	0.99	0.94-1.04	0.778
Salaried service	238	14	1513	86	0.99	0.95-1.04	0.704
Household wealth status (PCA[c])							
Poorest	148	27	401	73	Reference		
Lower middle	131	21	503	79	**1.09**	**1.02-1.16**	**0.009**
Middle	97	15	572	86	**1.17**	**1.09-1.26**	**<0.001**
Upper middle	110	10	1005	90	**1.23**	**1.15-1.32**	**<0.001**
Richest	78	11	604	89	**1.21**	**1.13-1.30**	**<0.001**
Health services knowledge							
Does *not* have knowledge of local hospital	101	19	434	81	Reference		
Has knowledge of local hospital	463	15	2651	85	**1.05**	**1.01-1.09**	**0.025**

For peer review only - http://bmjopen.bmj.com/site/about/guidelines.xhtml

Multivariate analyses with different models			
Mobility, adjusting for demographics (# of children and age of index child)	0.97	0.94-1.01	0.126
Mobility, adjusting for socioeconomics (education, occupation, and wealth)	0.98	0.95-1.02	0.308
Mobility, adjusting for demographics and socioeconomics	0.98	0.95-1.02	0.396

[a]Full vaccination coverage per EPI program includes 1 dose of BCG, 3 doses of polio, 3 doses of DPT, and 1 dose of measles vaccines.
[b]PR, Prevalence Ratio.
[c]Polychoric Principal Components Analysis was used to create a household wealth index including structural housing characteristics, cooking fuel, drinking water, and sanitation.

For peer review only

For peer review only - http://bmjopen.bmj.com/site/about/guidelines.xhtml

Table 4: Qualified provider visits for severe acute respiratory illness within prior year among children < 5 years old and association with mobility using univariate and multivariate models with modified Poisson regression

	No qualified provider for severe ARI[a] n=358 (25%)		Qualified provider for severe ARI[a] n=1100 (75%)		PR[b]	95% CI	p-value
Univariate analyses							
Mobility status	n	%	n	%			
Residentially stable ≥24 months	141	18	622	82	Reference		
Recently relocated ≤12 months	217	31	478	69	**0.84**	**0.79-0.90**	**<0.001**
Demographics	mean	SE	mean	SE			
Number of children <5yrs in household	1.23	0.03	1.25	0.02	1.02	0.97-1.08	0.437
Age of index child in months	29.1	0.83	23.7	0.44	**0.994**	**0.992-0.996**	**<0.001**
Socioeconomics							
Mother's education	n	%	n	%			
No education	134	38	220	62	Reference		
Some schooling	127	32	267	68	1.09	0.96-1.23	0.170
Finished secondary	79	16	425	84	**1.36**	**1.20-1.54**	**<0.001**
> Higher secondary	18	9	188	91	**1.47**	**1.30-1.66**	**<0.001**
Occupation of household head							
Unemployed or other	24	23	82	77	Reference		
Daily labor	139	36	244	64	**0.82**	**0.72-0.94**	**0.005**
Shopkeeper or merchant	89	22	320	78	1.01	0.90-1.14	0.848
Salaried service	106	19	454	81	1.05	0.93-1.18	0.435
Household wealth status (PCA[c])							
Poorest	154	40	228	60	Reference		
Lower middle	93	30	220	70	**1.18**	**1.02-1.37**	**0.031**
Middle	59	21	220	79	**1.32**	**1.16-1.51**	**<0.001**
Upper middle	37	12	277	88	**1.48**	**1.31-1.67**	**<0.001**
Richest	15	9	155	91	**1.53**	**1.35-1.73**	**<0.001**
Health services knowledge							
Does *not* have knowledge of local hospital	114	49	118	51	Reference		

For peer review only

For peer review only - http://bmjopen.bmj.com/site/about/guidelines.xhtml

Has knowledge of local hospital	244	20	982	80	**1.57**	**1.34-1.85**	**<0.001**
Multivariate analyses with different models							
Mobility, adjusting for demographics (# of children and age of index child)					**0.84**	**0.79-0.89**	**<0.001**
Mobility, adjusting for socioeconomics (education, occupation, and wealth)					**0.89**	**0.84-0.94**	**<0.001**
Mobility, adjusting for demographics and socioeconomics					**0.88**	**0.84-0.93**	**<0.001**

[a]ARI, Acute Respiratory Illness.
[b]PR, Prevalence Ratio.
[c]Polychoric Principal Components Analysis was used to create a household wealth index including structural housing characteristics, cooking fuel, drinking water, and sanitation.

For peer review only

For peer review only - http://bmjopen.bmj.com/site/about/guidelines.xhtml

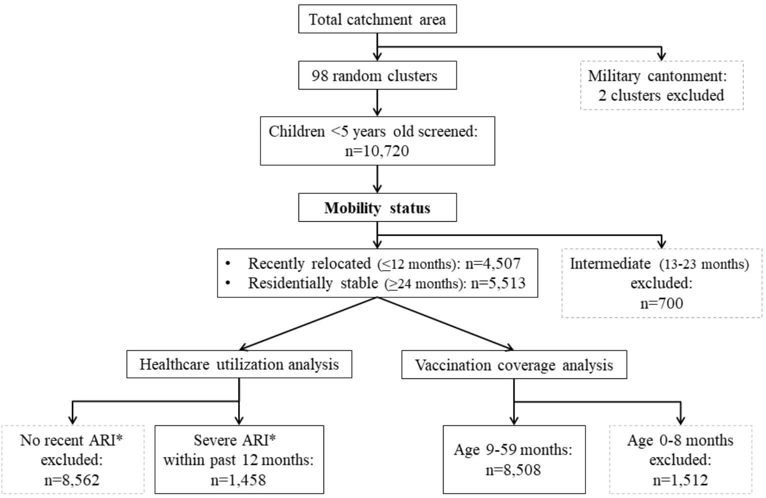

Figure 1: Children sampled by mobility status for healthcare utilization and vaccination coverage analysis
*ARI = Acute Respiratory Illness

Figure 1: Children sampled by mobility status for healthcare utilization and vaccination coverage analysis

338x190mm (96 x 96 DPI)

Effect of household relocation on child vaccination and health service utilization in Dhaka, Bangladesh: a cross-sectional community survey

SUPPLEMENTAL FIGURES AND TABLES

Supplemental Table 1: Household wealth principal components analysis coding and variable loading

Indicator	Coding	Loading
Number of rooms	Continuous	0.337
Housing arrangement	1 = Free<br>2 = Rental<br>3 = Owned	0.235
Roof	1 = Natural roof (bamboo/thatch)<br>2 = Rudimentary roof (tin)<br>3 = Finished roof (cement/concrete/tiled)<br>4 = Other	0.392
Walls	1 = Natural walls (jute/bamboo/mud)<br>2 = Rudimentary walls (wood)<br>3 = Finished walls (tin/brick/cement)<br>4 = Other	0.316
Floor	1 = Natural floor (earth/bamboo)<br>2 = Rudimentary floor (wood)<br>3 = Finished floor (cement/concrete)<br>4 = Other	0.377
Cooking fuel	1 = Natural (wood/grass/dung)<br>2 = Coal/charcoal<br>3 = Kerosene<br>4 = Electricity<br>5 = Gas (liquid/biogas)<br>6 = Other	0.350
Drinking water	1 = Unimproved (unprotected/surface/bottled)<br>2 = Improved but not piped<br>3 = Improved and piped into yard or private dwelling<br>4 = Other	0.170
Sanitation	1 = Unimproved (open defecation/hanging/open or broken pit)<br>2 = Improved but not piped	0.374

For peer review only

	3 = Improved and piped sewer<br>4 = Other	
Mobile phone ownership	0 = No<br>1 = Yes	0.380

Note: 51% of overall variance was explained by the first component.

For peer review only

For peer review only - http://bmjopen.bmj.com/site/about/guidelines.xhtml

Supplemental Table 2: ***Using EPI card plus maternal recall*****, vaccination coverage among children age 9-23 months and association with mobility status using univariate and multivariate models with modified Poisson regression**

	Partial vaccination		Full vaccination[a]		PR[b]	95% CI	p-value
	n=518 (21%)		n=1906 (79%)				
Univariate analyses							
Mobility status	n	%	n	%			
Residentially stable ≥24 months	243	19	1053	81	Reference		
Recently relocated ≤12 months	275	24	853	76	**0.93**	**0.88-0.98**	**0.009**
Demographics	mean	SE	mean	SE			
Number of children <5yrs in household	1.31	0.03	1.24	0.01	**0.94**	**0.89-0.99**	**0.035**
Age of index child in months	14.73	0.20	16.35	0.11	**1.02**	**1.02-1.03**	**<0.001**
Socioeconomics							
Mother's education	n	%	n	%			
No education	207	41	303	59	Reference		
Some schooling	121	22	433	78	**1.32**	**1.18-1.47**	**<0.001**
Finished secondary	141	16	719	84	**1.41**	**1.27-1.56**	**<0.001**
> Higher secondary	49	10	451	90	**1.52**	**1.36-1.69**	**<0.001**
Occupation of household head							
Unemployed or other	26	15	147	85	Reference		
Daily labor	195	38	318	62	**0.73**	**0.66-0.81**	**<0.001**
Shopkeeper or merchant	124	19	538	81	0.96	0.88-1.04	0.287
Salaried service	173	16	903	84	0.99	0.91-1.07	0.752
Household wealth status (PCA[c])							
Poorest	212	40	321	60	Reference		
Lower middle	114	25	348	75	**1.25**	**1.11-1.40**	**<0.001**
Middle	84	18	382	82	**1.36**	**1.20-1.54**	**<0.001**
Upper middle	59	10	560	90	**1.50**	**1.34-1.69**	**<0.001**
Richest	49	14	295	86	**1.42**	**1.26-1.61**	**<0.001**
Health services knowledge							
Does *not* have knowledge of local hospital	127	28	324	72	Reference		
Has knowledge of local hospital	391	20	1582	80	**1.12**	**1.05-1.19**	**<0.001**

For peer review only

Multivariate analyses with different models			
Mobility, adjusting for demographics (# of children and age of index child)	**0.93**	**0.88-0.98**	**0.007**
Mobility, adjusting for socioeconomics (education, occupation, and wealth)	0.96	0.91-1.01	0.136
Mobility, adjusting for demographics and socioeconomics	0.96	0.92-1.01	0.138

[a]Full vaccination coverage per EPI program includes 1 dose of BCG, 3 doses of polio, 3 doses of DPT, and 1 dose of measles vaccines.
[b]PR, Prevalence Ratio.
[c]Polychoric Principal Components Analysis was used to create a household wealth index including structural housing characteristics, cooking fuel, drinking water, and sanitation.

For peer review only

Supplemental Table 3: *Using EPI card only*, vaccination coverage among children age 9-23 months who have EPI cards and association with mobility status using univariate and multivariate models with modified Poisson regression

	Partial vaccination n=281 (19%)		Full vaccination[a] n=1222 (81%)		PR[b]	95% CI	p-value
Univariate analyses							
Mobility status	n	%	n	%			
Residentially stable ≥24 months	156	18	715	82	Reference		
Recently relocated ≤12 months	125	20	507	80	0.97	0.92-1.04	0.469
Demographics	mean	SE	mean	SE			
Number of children <5yrs in household	1.23	0.03	1.21	0.01	0.98	0.93-1.04	0.543
Age of index child in months	14.4	0.27	15.9	0.15	**1.02**	**1.01-1.03**	**<0.001**
Socioeconomics							
Mother's education	n	%	n	%			
No education	94	35	176	65	Reference		
Some schooling	57	17	286	83	**1.28**	**1.15-1.42**	**<0.001**
Finished secondary	93	17	461	83	**1.28**	**1.14-1.43**	**<0.001**
> Higher secondary	37	11	299	89	**1.37**	**1.22-1.52**	**<0.001**
Occupation of household head							
Unemployed or other	14	12	100	88	Reference		
Daily labor	89	33	184	67	**0.77**	**0.68-0.87**	**<0.001**
Shopkeeper or merchant	66	16	352	84	0.96	0.88-1.04	0.327
Salaried service	112	16	586	84	0.96	0.88-1.04	0.297
Household wealth status (PCA[c])							
Poorest	84	32	179	68	Reference		
Lower middle	67	24	211	76	1.12	0.99-1.25	0.062
Middle	52	17	248	83	**1.21**	**1.08-1.37**	**0.002**
Upper middle	42	10	381	90	**1.32**	**1.18-1.48**	**<0.001**
Richest	36	15	203	85	**1.25**	**1.10-1.41**	**<0.001**
Health services knowledge							
Does *not* have knowledge of local hospital	55	22	194	78	Reference		
Has knowledge of local hospital	226	18	1028	82	1.05	0.98-1.13	0.141

For peer review only - http://bmjopen.bmj.com/site/about/guidelines.xhtml

Multivariate analyses with different models				
Mobility, adjusting for demographics (# of children and age of index child)		0.98	0.93-1.04	0.582
Mobility, adjusting for socioeconomics (education, occupation, and wealth)		1.00	0.94-1.06	0.948
Mobility, adjusting for demographics and socioeconomics		1.00	0.95-1.07	0.888

[a]Full vaccination coverage per EPI program includes 1 dose of BCG, 3 doses of polio, 3 doses of DPT, and 1 dose of measles vaccines.
[b]PR, Prevalence Ratio.
[c]Polychoric Principal Components Analysis was used to create a household wealth index including structural housing characteristics, cooking fuel, drinking water, and sanitation.

For peer review only

For peer review only - http://bmjopen.bmj.com/site/about/guidelines.xhtml

Effect of household relocation on child vaccination and health service utilization in Dhaka, Bangladesh: a cross-sectional community survey

STROBE 2007 (v4) Statement—Checklist of items that should be included in reports of *cross-sectional studies*

Section/Topic	Item #	Recommendation	Reported on page #
**Title and abstract**	1	(*a*) Indicate the study's design with a commonly used term in the title or the abstract	1
		(*b*) Provide in the abstract an informative and balanced summary of what was done and what was found	2
**Introduction**			
Background/rationale	2	Explain the scientific background and rationale for the investigation being reported	4
Objectives	3	State specific objectives, including any prespecified hypotheses	4
**Methods**			
Study design	4	Present key elements of study design early in the paper	5
Setting	5	Describe the setting, locations, and relevant dates, including periods of recruitment, exposure, follow-up, and data collection	5
Participants	6	(*a*) Give the eligibility criteria, and the sources and methods of selection of participants	5
Variables	7	Clearly define all outcomes, exposures, predictors, potential confounders, and effect modifiers. Give diagnostic criteria, if applicable	7
Data sources/ measurement	8*	For each variable of interest, give sources of data and details of methods of assessment (measurement). Describe comparability of assessment methods if there is more than one group	7
Bias	9	Describe any efforts to address potential sources of bias	7
Study size	10	Explain how the study size was arrived at	6
Quantitative variables	11	Explain how quantitative variables were handled in the analyses. If applicable, describe which groupings were chosen and why	7
Statistical methods	12	(*a*) Describe all statistical methods, including those used to control for confounding	7
		(*b*) Describe any methods used to examine subgroups and interactions	7
		(*c*) Explain how missing data were addressed	7
		(*d*) If applicable, describe analytical methods taking account of sampling strategy	7

For peer review only

		(*e*) Describe any sensitivity analyses	8
**Results**			
Participants	13*	(a) Report numbers of individuals at each stage of study—eg numbers potentially eligible, examined for eligibility, confirmed eligible, included in the study, completing follow-up, and analysed	8
		(b) Give reasons for non-participation at each stage	8
		(c) Consider use of a flow diagram	Figure 1
Descriptive data	14*	(a) Give characteristics of study participants (eg demographic, clinical, social) and information on exposures and potential confounders	8
		(b) Indicate number of participants with missing data for each variable of interest	9
Outcome data	15*	Report numbers of outcome events or summary measures	9
Main results	16	(*a*) Give unadjusted estimates and, if applicable, confounder-adjusted estimates and their precision (eg, 95% confidence interval). Make clear which confounders were adjusted for and why they were included	9
		(*b*) Report category boundaries when continuous variables were categorized	8
		(*c*) If relevant, consider translating estimates of relative risk into absolute risk for a meaningful time period	n/a
Other analyses	17	Report other analyses done—eg analyses of subgroups and interactions, and sensitivity analyses	9
**Discussion**			
Key results	18	Summarise key results with reference to study objectives	10
Limitations	19	Discuss limitations of the study, taking into account sources of potential bias or imprecision. Discuss both direction and magnitude of any potential bias	11
Interpretation	20	Give a cautious overall interpretation of results considering objectives, limitations, multiplicity of analyses, results from similar studies, and other relevant evidence	12
Generalisability	21	Discuss the generalisability (external validity) of the study results	13
**Other information**			
Funding	22	Give the source of funding and the role of the funders for the present study and, if applicable, for the original study on which the present article is based	14

*Give information separately for cases and controls in case-control studies and, if applicable, for exposed and unexposed groups in cohort and cross-sectional studies.

Note: An Explanation and Elaboration article discusses each checklist item and gives methodological background and published examples of transparent reporting. The STROBE checklist is best used in conjunction with this article (freely available on the Web sites of PLoS Medicine at http://www.plosmedicine.org/, Annals of Internal Medicine at http://www.annals.org/, and Epidemiology at http://www.epidem.com/). Information on the STROBE Initiative is available at www.strobe-statement.org.

For peer review only

For peer review only

Effect of household relocation on child vaccination and health service utilization in Dhaka, Bangladesh: a cross-sectional community survey

Journal:	*BMJ Open*
Manuscript ID	bmjopen-2018-026176.R1
Article Type:	Research
Date Submitted by the Author:	16-Jan-2019
Complete List of Authors:	Horng, Lily; Stanford University, Infectious Diseases and Geographic Medicine<br>Kakoly, Nadira Sultana; International Centre for Diarrheal Disease Research, Bangladesh (icddr, b)<br>Abedin, Jaynal ; International Centre for Diarrheal Disease Research, Bangladesh (icddr, b)<br>Luby, Stephen; Stanford University, Infectious Diseases and Geographic Medicine; International Centre for Diarrheal Disease Research, Bangladesh (icddr, b)
**Primary Subject Heading**:	Global health
Secondary Subject Heading:	Epidemiology, Paediatrics, Public health, Infectious diseases
Keywords:	Community child health < PAEDIATRICS, PUBLIC HEALTH, Public health < INFECTIOUS DISEASES, Paediatric infectious disease & immunisation < PAEDIATRICS, EPIDEMIOLOGY

SCHOLARONE™ Manuscripts

Effect of household relocation on child vaccination and health service utilization in Dhaka, Bangladesh: a cross-sectional community survey

Lily M. Horng[1]*, Nadira K. Sultana[2], Jaynal Abedin[2], Stephen P. Luby[1, 2]

1 Division of Infectious Diseases & Geographic Medicine, Department of Medicine, Stanford University, Stanford, California, 94305, USA

2 International Centre for Diarrheal Disease Research, Bangladesh (icddr, b), Dhaka 1000, Bangladesh

*Corresponding author:

Lily M. Horng, MD MPH

Division of Infectious Diseases and Geographic Medicine,

Stanford University, Stanford, California, 94305, USA

+16507212730

lhorng@stanford.edu

Word count (excluding title page, abstract, references, figures and tables): 4,309 words

For peer review only - http://bmjopen.bmj.com/site/about/guidelines.xhtml

Abstract

Objective To explore the relationship between household relocation and use of vaccination and health services for severe acute respiratory illness (ARI) among children in Dhaka, Bangladesh.

Design Analysis of cross-sectional community survey data from a prior study examining the impact of *Haemophilus influenzae* type b vaccine introduction in 2009 on meningitis incidence in Bangladesh.

Setting Communities surrounding two large pediatric hospitals in Dhaka, Bangladesh.

Participants Households with children under 5 years old who either recently relocated $\leq$ 12 months or who were residentially stable living $\geq$ 24 months in their current residence (total n = 10,020) were selected for this study.

Primary outcome measures Full vaccination coverage among 9-59 month old children and visits to a qualified medical provider for severe ARI among children under 5 years old.

Results Using vaccination cards with maternal recall, full vaccination was 80% among recently relocated children (n=3,795) and 85% among residentially stable children (n=4,713; χ^2=37.2, p<0.001). Among children with ARI in the prior year, 69% of recently relocated children (n=695) had visited a qualified provider compared to 82% of residentially stable children (n=763; χ^2=31.9, p<0.001). After adjusting for demographic and socioeconomic characteristics, recently relocated children were less likely to be fully vaccinated (prevalence ratio [PR] 0.97; 95% confidence interval [CI] 0.95-0.99; p=0.016) and to have visited a qualified provider for ARI (PR 0.88; 95% CI 0.84-0.93; p<0.001).

Conclusions Children in recently relocated households in Dhaka, Bangladesh have decreased use of vaccination and qualified health services for severe acute respiratory illnesses.

Strengths and limitations of this study

For peer review only - http://bmjopen.bmj.com/site/about/guidelines.xhtml

- This study examined a rich dataset from prior community surveys in Dhaka, Bangladesh to explore associations between household relocation and utilization of vaccination and qualified child health services.
- Vaccination was evaluated using different measurements and age ranges to explore trends in the relationship between mobility and vaccination.
- Effect of household relocation on use of child health services was found even after adjusting for socioeconomic factors known to impact health-seeking behavior.
- Limitations include lack of detailed data on mobility patterns and costs of health services.

For peer review only

For peer review only - http://bmjopen.bmj.com/site/about/guidelines.xhtml

Introduction

Pneumonia or acute respiratory illness (ARI) is the leading cause of death globally in children under 5 years old, and lower respiratory tract infections caused an estimated 652,572 child deaths in 2016.[1] Many causes of ARI are preventable by vaccines such as *Streptococcus pneumoniae* (attributed to 52% of global pneumonia child deaths) and *Haemophilus influenzae* type b (Hib) (7% of deaths).[1] The majority of the world's population now lives in urban areas, and this population is expected to grow from 54% in 2014 to 66% in 2050, an estimated increase of 2.5 billion people.[2,3] With this increase, 90% of growth is projected in Asia and Africa.[3] The population living in urban slums is also expected to increase from 881 million in 2014 to 2 billion in 2030, in large part due to rural-urban migration.[2,4]

In many low- and middle-income countries, vaccination and childhood mortality rates among urban poor are worse than among other urban groups and even rural populations.[5,6] In addition, residents of slums have poor health outcomes due to lack of reliable access to housing, clean water, sanitation, education, and health services.[4,5,7–9] In Nigeria, a 2010 study examining 2003 Demographic and Health Survey data of 6,029 children 12 months and older found full immunization among 24.3% of rural non-migrant, 15.2% of urban non-migrant, and 8.5% of rural-urban migrant children.[10] In Bangladesh, a comparison study of the 2013 Urban Health Survey and 2014 Demographic and Health Survey found under-5 child mortality rates of 46 per 1,000 livebirths nationally, 41 in Dhaka, 49 in rural areas, and 57 in urban slums.[5] One recent systematic review found community factors associated with vaccination coverage in the urban poor included socio-economic characteristics, vaccination knowledge and beliefs, access to care, and recent rural-urban migration.[6]

Residential mobility has been recognized as an important contributor to healthcare use in high-income countries, with relocation associated with decreased use of preventive and curative services.[11,12] One study using a 1998 United States national health survey found that duration, distance, and frequency of moving were all predictors of decreased use of child

For peer review only - http://bmjopen.bmj.com/site/about/guidelines.xhtml

health services even after accounting for sociodemographic factors. Households who had moved within 12 months accessed fewer preventive child health services compared to households living in their current residence over 36 months (odds ratio 3.1, 95% confidence interval [CI] 2.5-3.7).[12] Recently relocated households also accessed fewer curative services (odds ratio 3.3, 95% CI 2.6-4.2).[12] Frequent moving also impacted children's long-term cognitive function and behavioral problems into adulthood.[13]

Few studies examine mobility and healthcare utilization in low- and middle-income countries despite high population relevance: approximately 43% of urban residents in middle-income countries and 78% in low-income countries live in slums.[3,4,6,10,14,15] In the 2010 Nigeria study, urban non-migrant children had 1.7 times higher odds of being fully immunized than rural-urban migrants (univariate odds ratio [OR] 1.67, 95% confidence interval [CI] 1.20-2.32).[10] This association between migration and immunization was independent of demographic factors, but was attenuated and partially explained by socioeconomic characteristics and maternal healthcare utilization in multivariable analyses.[10] In this Nigeria study, migrant status was defined as moving within 10 years.[10] A 2010 cross-sectional survey in India examined 746 rural-urban migrant mothers with children under 2 years old: 339 were 'recent' migrants who moved to Delhi within 5 years and 407 were 'settled' migrants in Delhi at least 5 years.[15] For age-appropriate children, 81% of settled migrants and 64% of recent migrants were fully immunized per national guidelines.[15] Settled migrant children had 1.9 times higher odds of being fully immunized than recent migrants after adjusting for demographics, socioeconomics, and maternal healthcare utilization (adjusted OR 1.93, 95% CI 1.18-3.14).[15]

Studying urban health services in Bangladesh is useful because Bangladesh is the world's most densely populated country that is not a city-state: the population of the capital Dhaka will increase from an estimated 16 to 27 million by 2030.[3,16] Furthermore, the government has a strong national Expanded Programme on Immunization and active health systems research.[16,17] In 2011, full vaccination rates among children age 12-23 months

For peer review only - http://bmjopen.bmj.com/site/about/guidelines.xhtml

were 80% nationally in Bangladesh, 75% in Dhaka, but only 43-67% in Dhaka slums.[16–20] Prior studies found that household turnover was as high as 50% in one year, comprehensive provider-led vaccination interventions were effective but too expensive to sustain, and street children were very hard to reach with interventions in Dhaka.[18,21,22]

To explore the relationship between residential mobility and healthcare utilization in Dhaka, we used data from a study showing Hib vaccine introduction into Bangladesh's Expanded Programme on Immunization in 2009 dramatically reduced rates of Hib meningitis and purulent meningitis in children.[23] We conducted secondary analysis of the Hib impact study's community survey data to determine whether recently relocated children were: 1) less likely to be fully vaccinated per Expanded Programme on Immunization guidelines and 2) less likely to use qualified health services for severe acute respiratory illness than residentially stable children.

Methods

Study design and setting

Hib conjugate vaccine was introduced into Bangladesh's Expanded Programme on Immunization in 2009, and the Hib impact study conducted pre and post-vaccine surveillance of meningitis in children under 5 years old using hospital records and community surveys surrounding two large pediatric hospitals in Dhaka: Dhaka Shishu and Shishu Shastya Foundation Hospital.[23] Field researchers consecutively enrolled 100 children discharged with a diagnosis of meningitis and/or encephalitis from the two study hospitals, visited households, and recorded household geographical positional system coordinates. The catchment area was defined as the area containing >80% of households with children discharged with meningitis and within one hour of transport to either hospital. Field teams divided the catchment area into 1,748 equal-sized rectangles and randomly selected 100 rectangles as clusters. Teams surveyed each household with a child under 5 years old within

For peer review only - http://bmjopen.bmj.com/site/about/guidelines.xhtml

98 clusters. Two clusters were within a military cantonment, thus inaccessible. Households were asked about: 1) routine vaccinations using vaccination cards and maternal recall and 2) healthcare use for children with illnesses in the prior 12 months suggestive of meningoencephalitis defined as: any serious illness with acute onset of fever with either convulsions or unconsciousness or altered mental status.[23] Data were collected one year before (2008) and after (2010) Hib vaccine introduction.

Study population

We used the Hib impact study's pre-vaccine community surveillance data and included children based on mobility status: 1) children living in their current residence ≤ 12 months who we classified as "recently relocated" and 2) children living ≥ 24 months in their current residence who we classified as "residentially stable". This definition of mobility/migration status has been used in prior studies.[6,12] We excluded children living in their current residence 13-23 months who we classified as "intermediately mobile".

Study outcomes

Our two primary outcomes focused on healthcare utilization: 1) full vaccination among 9-59 month old children and 2) visit to a qualified medical provider among children under 5 years old who had severe acute respiratory illness symptoms within the prior 12 months. We defined full vaccination per Bangladesh Expanded Programme on Immunization guidelines in 2008 (before Hib vaccine): 1 dose of Bacillus Calmette-Guérin (BCG) vaccine against tuberculosis; 3 doses of combined vaccine against diphtheria, pertussis, and tetanus (DPT); 3 doses of oral polio vaccine (excluding polio vaccine given at birth); and 1 dose of measles vaccine. Government guidelines recommended children to receive all these vaccinations before 9 months of age. Any doses of pentavalent vaccine, which includes diphtheria, pertussis, tetanus, hepatitis B, and Hib, were included in vaccination analyses. We defined severe acute respiratory illness as cough or difficulty breathing plus any danger sign:

For peer review only - http://bmjopen.bmj.com/site/about/guidelines.xhtml

stridor, chest in-drawing, difficulty drinking/ breastfeeding, vomiting, cyanosis, convulsions, lethargy, or unconsciousness. We defined a qualified medical provider as having a Bachelor of Medicine degree or higher.

Data analysis

We compared sociodemographic and health characteristics between residentially stable and recently relocated households. For continuous variables, we calculated means with standard errors and t-tests adjusting for cluster. For categorical variables, we calculated percentages and χ^2-tests. To construct a wealth index, we used polychoric principal components analysis (PCA) including: housing (number of rooms; free, rental, or owned housing; main material of roof, walls, and floors), cooking fuel, drinking water, sanitation, and mobile phone ownership.[24–26] We then divided households into wealth quintiles. We did not include durable assets such as furniture items because ownership of these goods could be associated with duration of residency.

To examine the magnitude of association between mobility and study outcomes of vaccination and visit to a qualified provider for severe ARI, we used modified Poisson regression with robust cluster variance to estimate prevalence ratios (PRs).[27,28] We chose modified Poisson regression to model prevalence ratios for common binary outcomes because logistic regression is more applicable to rare outcomes and because log-binomial regression models may fail to converge. We conducted univariate analyses to estimate individual effects of mobility, demographics, socioeconomics, and health services knowledge (i.e. knowledge of local hospital) on healthcare utilization. Missing data regarding main study outcome of acute respiratory illness were handled through listwise deletion. Given large number of missing vaccination cards, we analyzed vaccination in two ways: 1) using vaccination cards plus maternal recall and 2) using vaccination cards alone. We conducted multivariable analyses examining the association between mobility and healthcare utilization, adjusting for demographics and socioeconomics known to influence health-seeking behavior.[9,15,29–31]

For peer review only - http://bmjopen.bmj.com/site/about/guidelines.xhtml

Regression diagnostics included checks for influential observations with Cook's distance calculations.

Ethics

The Ethical Review Committee of the International Centre for Diarrhoeal Disease Research, Bangladesh (icddr,b) reviewed and approved the study protocol. Written informed consent was obtained from all participants before taking part in the initial Hib impact study.

Patient and public involvement

No participants were directly involved in development of the research questions and outcomes. No participants were involved in the design or conduct of the study. There are no plans to disseminate the results of the research individually to study participants.

Results

We surveyed a total of 10,720 households with children less than 5 years old: 42% of households had recently relocated within 12 months, 51% were residentially stable living in their current residence over 24 months, and 7% were intermediately mobile (Figure 1). We excluded from subsequent analyses 700 children living in their current residence 13-23 months and classified as intermediately mobile. For the healthcare utilization analysis, 1,458 children had severe ARI symptoms within the 12 months prior to survey. For the vaccination analysis, 8,508 children were age 9-59 months and thus should have completed all Expanded Programme on Immunization-recommended vaccinations. Household demographics, parental education, occupation, and hospital knowledge were available for all households. Missing data included: income for 12 households, meningitis symptoms for 3 children, respiratory illness symptoms for 1 child, and vaccination cards for 4,859 children age 9-59 months.

For peer review only - http://bmjopen.bmj.com/site/about/guidelines.xhtml

Recently relocated families had smaller households, less education, less wealth, and less knowledge of the local hospital compared to residentially stable families (Table 1). Recently relocated families were poorer: 24% earned less than 5,000 Bangladeshi taka (US $73) per month compared to 18% of residentially stable families. For the wealth index analysis, the first principal component accounted for 51% of overall variance, with largest contributions from roof and floor materials, sanitation, and mobile phone ownership (Supplemental Table 1). Among recently relocated families, 48% were in the two poorest wealth quintiles compared to 37% of residentially stable families. Fewer recently relocated caregivers had knowledge of the local hospital, 76%, compared to residentially stable caregivers, 85% (χ^2=142.3, p<0.001). Similar rates of illness in the 12 months prior to survey were reported by all households: 14-15% of children with symptoms of severe ARI and 3-4% with symptoms of meningitis/encephalitis.

Full vaccination coverage measured by vaccination card plus maternal recall was 83% among all children age 9-59 months (Table 2). Full vaccination was 80% among recently relocated children and 85% among residentially stable children (univariate PR 0.94, 95% CI 0.91-0.97, p<0.001). Vaccination was lower in households with more children and younger children. Socioeconomic factors, especially mother's education, had the strongest association with vaccination. In multivariable analyses, recently relocated children were 3% less likely than residentially stable children to be fully vaccinated even after adjusting for demographic and socioeconomic factors (multivariable PR 0.97, 95% CI 0.95-0.99, p=0.016).

Vaccination was also analyzed using only vaccination cards (Table 3). At time of survey, only 43% of all children had vaccination cards available. Fewer recently relocated children had vaccination cards, 36%, compared to 48% of residentially stable children (χ^2=126.9, p<0.001). Younger children were more likely to have cards than older children: 62% of children 9-23 months old had vaccination cards as compared to 38% of children 24-59 months old (χ^2=505.7, p<0.001). Full vaccination per vaccination card was 83% among recently relocated children and 86% among residentially stable children (univariate PR 0.97,

For peer review only

For peer review only - http://bmjopen.bmj.com/site/about/guidelines.xhtml

95% CI 0.93-1.00, p=0.083). The 9-59 month age range for vaccination analysis allowed inclusion of a larger sample size of children vulnerable to vaccine-preventable disease. In contrast, routine vaccination schedules focus on children <2 years old and full vaccination coverage per Expanded Programme on Immunization in Bangladesh and many other countries is defined in children up to 23 months old.[17,32] Narrowing the age range of vaccination analysis to 9-23 months showed similar results although with smaller sample sizes limiting statistical power to detect mobility-vaccination associations (Supplementary Tables 2 and 3). In addition, using a 10-month age minimum to account for potential delay in measles vaccination recommended at 9 months old showed the same results as a 9-month age cutoff (data not shown). Checking for influential observations with Cook's distances identified no outliers in vaccination analyses (Supplemental Figure 1).[33]

Among all children under 5 years old with severe ARI in the past year, 75% visited a qualified medical provider (Table 4). Fewer recently relocated children with severe ARI saw a qualified provider, 69%, as compared to 82% of residentially stable children (univariate PR 0.84, 95% CI 0.79-0.90, p<0.001). Socioeconomic factors, especially household wealth, were strongly associated with qualified provider visits.

Health services knowledge was also strongly associated with acute healthcare visits: 80% of parents who knew about the local hospital sought ARI treatment from a qualified provider as compared to 51% of parents who did *not* have knowledge of the local hospital (univariate PR 1.57, 95% CI 1.34-1.85, p<0.001). After adjusting for demographic and socioeconomic factors, recently relocated households were 11% less likely than residentially stable households to visit a qualified medical provider for children with severe ARI (multivariable PR 0.88, 95% CI 0.84-0.93, p<0.001). One outlier was identified by Cook's distances in healthcare utilization analyses (Supplemental Figure 1). Excluding this outlier and using robust error variance resulted in similar results to those presented (data not shown).

Discussion

For peer review only - http://bmjopen.bmj.com/site/about/guidelines.xhtml

Recently relocated households were less likely to use both acute and preventive child healthcare services in our study in Dhaka, Bangladesh, and these findings support prior literature exploring the effects of mobility on healthcare utilization.[6,10–13,15] Household relocation had a strong association with decreased use of qualified medical services for severe acute respiratory illness. Similarly, household relocation was associated with decreased vaccination rates although this relationship was less robust. Another key finding was that recently relocated parents were less knowledgeable about the local hospital compared to residentially stable parents, and knowledge of the local hospital had as strong an association with acute healthcare visits as some economic factors. Overall, recently relocated children in our study had slightly lower vaccination rates and markedly lower use of acute healthcare services for ARI than residentially stable children.

Study strengths include data focused on urban Bangladesh and exploring vaccination status using different measurements as well as adjusting for socioeconomic factors when examining mobility and health service utilization. Our study used the Hib impact study's rigorous community surveillance data of households living close to tertiary care pediatric hospitals in Dhaka. Dhaka residents have high mobility and many options for healthcare. Unlike in rural areas, physical access to health services is usually *not* a barrier to healthcare use in urban areas. One study found almost all residents in Dhaka lived within 1 kilometer of primary health services.[34] Routine immunizations are provided free by the government of Bangladesh, but acute care services require out of pocket expenditures which can be a barrier to access. Our findings on mobility and child health services use in Dhaka could inform health services work in other urban low- and middle-income country contexts.[3,5]

We analyzed vaccination using vaccination card data augmented with maternal recall, vaccination card data only, and several different age ranges. Accurately measuring vaccinations in children is a known difficulty in public health programs and research studies, especially in low- and middle-income countries where vaccination cards are frequently *not* available. Some studies have found poor agreement between parental recall, vaccination

For peer review only - http://bmjopen.bmj.com/site/about/guidelines.xhtml

cards, and even official health records.[35–37] By contrast, other studies have found good correlation between maternal report and vaccination cards, and maternal recall is routinely used in Demographic and Health Surveys and Multiple Indicator Cluster Surveys.[38,39] Maternal recall can overestimate or underestimate vaccination history based on education, social desirability bias, and vaccine-specific knowledge. Vaccination card retention itself can be affected by parental education, household wealth, age of child, and even household relocation. Our data showed more missing cards among recently relocated households and in older children, thus those groups are subject to more maternal recall bias. Using only vaccination cards or narrower age ranges in our vaccination analyses resulted in smaller sample sizes which limited statistical power, but all analyses showed similar effect estimates of increased mobility associated with decreased vaccination. Moreover, the association between increased household relocation and decreased health services use was still seen even after adjusting for socioeconomic factors known to impact healthcare use.

Study limitations include lack of data on mobility patterns and health services costs. Information on households' prior residences, distances moved, or frequency of moving was not available in our dataset. Households moving from rural Bangladesh to urban Dhaka, moving long distances, or relocating frequently probably have less knowledge and therefore use of locally available health services.[12] Several studies show that recent rural to urban migration is associated with lower vaccination coverage in children.[6,10,15] Lack of data on mobility patterns in our study precludes evaluation of how magnitude of relocation affected healthcare use. Our findings likely underestimate the negative association between mobility and healthcare utilization for households with large migration such as rural to urban migrants as compared to households with intra-Dhaka relocation where one would expect minimal change in health-seeking behavior. We were also unable to examine household relocation timing in relation to healthcare use. Recently relocated households were not asked if healthcare visits occurred before or after moving. Healthcare visits before moving would not be relevant to how mobility affects use of health services after moving. Our findings may

For peer review only - http://bmjopen.bmj.com/site/about/guidelines.xhtml

underestimate the negative association between mobility and health-seeking behavior because our data included healthcare visits before moving which were unrelated to knowledge of the new geographic area. Timing of healthcare visits in relation to acquiring knowledge of hospital services was also not available, thus there may be reverse causality of recently relocated households gaining knowledge of local providers after seeking care. Ultimately, our results show a modest overall association between mobility and healthcare use which could be elucidated by asking about migration patterns including timing of use of health services.

Our study results may not be as generalizable to populations in urban areas without tertiary care hospitals. Our sampling scheme focused on community catchment areas surrounding tertiary care pediatric hospitals. Advantages of this study design were that it allowed examination of healthcare utilization for severe disease since advanced services were available within a small physical distance. In addition, it was a low-cost way to examine population-level mobility instead of more resource-intensive active surveillance of migrant populations. However, use of health services generally increases with geographic proximity, and studies show this relationship is influenced by many factors including income and slum versus non-slum locations.[34,40] Recently relocated populations may be even more influenced by proximity than residentially stable populations because of fewer socioeconomic resources and lack of knowledge of health services. This would bias our results towards higher rates of health-seeking behavior among recently relocated households. Recently relocated households in areas without tertiary care services may use health services less because of transport costs and lack of knowledge of health facilities physically distant. In addition, our study included participants who relocated into the study area, but not people who left the study area. In-migrants and out-migrants may be different in their healthcare utilization patterns, which could also affect generalizability.

Our dataset did not contain cost of services, which is a well-known barrier to healthcare use.[19,20,30] Although vaccinations are provided for free, some non-governmental and private organizations charge fees for patient registration. Even small fees

For peer review only - http://bmjopen.bmj.com/site/about/guidelines.xhtml

could have lowered vaccination rates. Cost of services, willingness to pay, and underlying finances are strongly linked, thus adjusting for socioeconomic factors of parental education and wealth in our models should have incorporated some cost effects on healthcare use. However, costs could affect recently relocated households disproportionately more than residentially stable households of the same socioeconomic status. One could hypothesize that immediately after relocating, families would first spend money on household goods before preventive medicine fees. Without cost data, we can still conclude from our analysis that increased mobility is associated with decreased healthcare use, but we have limited understanding of mechanisms through which mobility affects healthcare use.

Barriers and delays to using appropriate healthcare services increase mortality.[30,41] One study in India of 290 children hospitalized for pneumonia in a tertiary care center found that delayed hospital referral, defined as three or more days between symptom onset and hospitalization, was associated with increased mortality (OR 52.1, 95% CI 6.7-402.4, $p<0.001$) after adjusting for age, residence in slum, and illness severity.[41] In this study, incomplete immunization was also associated with increased mortality (OR 12.3, 95% CI 2.2-69.9, $p=0.005$).[41] Reasons for delayed care-seeking can include access and cost. While cost does influence healthcare use, parents of sick children usually do seek some treatment. In the 2014 Bangladesh Demographic and Health Survey, 52% of urban parents with children with ARI symptoms in the 2 weeks prior to survey sought treatment from a health facility, 23% went to pharmacies, and 12% went to traditional practitioners.[42] Only 12% of parents with sick children sought no health care treatment at all.[42] While cost does not seem a large barrier to seeking any treatment at all, cost likely influences choice of health provider.

Household relocation disrupts prior relationships with healthcare providers and results in lack of familiarity with local services. A World Health Organization (WHO) conceptual framework on social determinants of health can explore how mobility relates to healthcare use.[43] In the WHO framework, structural determinants of social, economic, and political contexts influence intermediary determinants of material, behavioral, psychosocial,

For peer review only - http://bmjopen.bmj.com/site/about/guidelines.xhtml

and health system factors that ultimately determine an individual's health. Relocation can improve an individual's socioeconomic position in the long term, but mobility often disrupts material resources, psychosocial support, and health care access. Studies show that continuity of care is associated with increased vaccination, fewer emergency department visits, and decreased hospitalization among children.[12,44–46] People usually move to new areas because of pre-existing social connections through family, friends, or work.[7,47] These social contacts can act as pathways of important local knowledge, including health services, but recently relocated households have fewer social contacts and access fewer information sources. Other studies have also found that parental attitudes and knowledge are critical factors contributing to use of health services.[6,48–51] One literature review found that *practical* knowledge about vaccination schedule, timing, and logistics had a stronger association with vaccination uptake than scientific knowledge of vaccine names or biologic actions.[50] One study in India of 210 residents in slums and 100 migrant families of construction workers found 28% of slum residents and 64% of migrants identified lack of knowledge of place or time of services as a reason for decreased immunization.[51] Our study did not ask specifically about knowledge of vaccination services, but future research on knowledge of services could help elucidate how to connect new migrants to care.

Our finding that recently relocated children in Dhaka use fewer qualified health services compared to residentially stable children sheds light on health barriers faced by a growing population of children living in urban centers of low- and middle-income countries. Rigorous community surveillance in hospital catchment areas allows for increased understanding of factors affecting access to and use of healthcare services. Policymakers working to improve urban child health could invest in accurate counting of children living in communities with high household turnover in order to connect recently relocated households to already existing local health services. Further studies by researchers on patterns and mechanisms through which mobility affects healthcare use could inform critical intervention

For peer review only - http://bmjopen.bmj.com/site/about/guidelines.xhtml

points. Ultimately, cost-effective and targeted interventions to increase appropriate healthcare use among recently relocated children could improve health of future urban populations.

Contributors

SL was the Principal Investigator and involved in every aspect of the study from conceptualization to data analysis to manuscript writing and submission. NS, JA, and LH were involved in data analysis, and LH drafted the written work. All authors collaborated on and approved the final manuscript.

Competing interests

None declared.

Provenance and peer review

Not commissioned; externally peer reviewed.

Ethical considerations

The Ethical Review Committee of the International Centre for Diarrhoeal Disease Research, Bangladesh (icddr,b) reviewed and approved the study protocol.

Participant consent

Written informed consent was obtained from all participants before taking part in the initial Hib impact study.

Funding

The original Hib impact study data collection was funded by the Bill and Melinda Gates Foundation and the GAVI Hib Initiative (GR-00580). No additional funding was provided for this study.

For peer review only - http://bmjopen.bmj.com/site/about/guidelines.xhtml

Data sharing

Data are available by emailing the corresponding author LH at lhorng@stanford.edu.

Acknowledgements

The authors thank study participants, field researchers, and collaborative partners of the Hib impact study who included Dhaka Shishu microbiology laboratories and the Johns Hopkins Bloomberg School of Public Health. icddr,b is grateful to the Bangladesh, Canada, Sweden and the United Kingdom governments for providing core/unrestricted support.

For peer review only

References

1 Troeger C, Blacker B, Khalil IA, *et al.* Estimates of the global, regional, and national morbidity, mortality, and aetiologies of lower respiratory infections in 195 countries, 1990–2016: a systematic analysis for the Global Burden of Disease Study 2016. *Lancet Infect Dis* 2018;**18**:1191–210. doi:10.1016/S1473-3099(18)30310-4

2 UN-Habitat. *World Cities Report 2016: Urbanization and Development – Emerging Futures*. New York: UN-Habitat 2016. https://unhabitat.org/books/world-cities-report/ (accessed 12 Jan 2019).

3 United Nations, Department of Economic and Social Affairs, Population Division. World Urbanization Prospects: The 2014 Revision [Highlights]. New York: United Nations 2015. http://esa.un.org/unpd/wup/Publications/Files/WUP2014-Highlights.pdf (accessed 3 Feb 2016).

4 UN-Habitat. *The challenge of slums: global report on human settlements 2003*. New York: UN-Habitat 2003.

5 Ezeh A, Oyebode O, Satterthwaite D, *et al.* The history, geography, and sociology of slums and the health problems of people who live in slums. *The Lancet* 2017;**389**:547–58. doi:10.1016/S0140-6736(16)31650-6

6 Crocker-Buque T, Mindra G, Duncan R, *et al.* Immunization, urbanization and slums – a systematic review of factors and interventions. *BMC Public Health* 2017;**17**:556. doi:10.1186/s12889-017-4473-7

7 Rashid SF. Strategies to reduce exclusion among populations living in urban slum settlements in Bangladesh. *J Health Popul Nutr* 2009;**27**:574–86.

8 UN-Habitat. *State of the World's Cities 2006/7*. New York: UN-Habitat 2006.

9 Unger A. Children's health in slum settings. *Arch Dis Child* 2013;**98**:799–805. doi:10.1136/archdischild-2011-301621

10 Antai D. Migration and child immunization in Nigeria: individual-and community-level contexts. *BMC Public Health* 2010;**10**:116.

11 Jelleyman T, Spencer N. Residential mobility in childhood and health outcomes: a systematic review. *J Epidemiol Community Health* 2008;**62**:584–92.

12 Fowler MG, Simpson GA, Schoendorf KC. Families on the move and children's health care. *Pediatrics* 1993;**91**:934–40.

13 Wood D, Halfon N, Scarlata D, *et al.* Impact of family relocation on children's growth, development, school function, and behavior. *JAMA* 1993;**270**:1334–8.

14 Awoh AB, Plugge E. Immunisation coverage in rural–urban migrant children in low and middle-income countries (LMICs): a systematic review and meta-analysis. *J Epidemiol Community Health* 2016;**70**:305–11.

15 Kusuma YS, Kumari R, Pandav CS, *et al.* Migration and immunization: determinants of childhood immunization uptake among socioeconomically disadvantaged migrants in Delhi, India. *Trop Med Int Health* 2010;**15**:1326–32.

For peer review only - http://bmjopen.bmj.com/site/about/guidelines.xhtml

16 NIPORT, icddr,b, MEASURE Evaluation. Bangladesh Urban Health Survey 2013 Final Report. 2015. http://www.cpc.unc.edu/measure/resources/publications/tr-15-117 (accessed 3 Feb 2016).

17 Government of Bangladesh. Bangladesh EPI coverage evaluation survey 2011, Expanded Programme on Immunization. 2011. http://www.dghs.gov.bd/images/docs/EPI/CES%20Reports/EPI%20Coverage%20Evaluation%20Survey%202011.pdf

18 Hayford K, Uddin MJ, Koehlmoos TP, *et al.* Cost and sustainability of a successful package of interventions to improve vaccination coverage for children in urban slums of Bangladesh. *Vaccine* 2014;**32**:2294–9. doi:10.1016/j.vaccine.2014.02.075

19 Uddin MJ, Larson CP, Oliveras E, *et al.* Child immunization coverage in urban slums of Bangladesh: impact of an intervention package. *Health Policy Plan* 2010;**25**. doi:10.1093/heapol/czp041

20 Perry H, Weierbach R, Hossain I, *et al.* Childhood immunization coverage in zone 3 of Dhaka City: the challenge of reaching impoverished households in urban Bangladesh. *Bull World Health Organ* 1998;**76**:565–73.

21 Luby SP, Halder AK, Saha SK, *et al.* A low-cost approach to measure the burden of vaccine preventable diseases in urban areas. *Vaccine* 2010;**28**:4903–12. doi:10.1016/j.vaccine.2010.05.040

22 Uddin MJ, Shamsuzzaman M, Horng L, *et al.* Use of mobile phones for improving vaccination coverage among children living in rural hard-to-reach areas and urban streets of Bangladesh. *Vaccine* 2016;**34**:276–83. doi:10.1016/j.vaccine.2015.11.024

23 Sultana NK, Saha SK, Al-Emran HM, *et al.* Impact of introduction of the Haemophilus influenzae type b conjugate vaccine into childhood immunization on meningitis in Bangladeshi infants. *J Pediatr* 2013;**163**:S73–8.

24 Vyas S, Kumaranayake L. Constructing socio-economic status indices: how to use principal components analysis. *Health Policy Plan* 2006;**21**:459–68. doi:10.1093/heapol/czl029

25 Luby SP, Halder AK. Associations among handwashing indicators, wealth, and symptoms of childhood respiratory illness in urban Bangladesh. *Trop Med Int Health* 2008;**13**:835–44. doi:10.1111/j.1365-3156.2008.02074.x

26 Sharker MY, Nasser M, Abedin J, *et al.* The risk of misclassifying subjects within principal component based asset index. *Emerg Themes Epidemiol* 2014;**11**:6.

27 Zou G. A modified poisson regression approach to prospective studies with binary data. *Am J Epidemiol* 2004;**159**:702–6.

28 Chen W, Shi J, Qian L, *et al.* Comparison of robustness to outliers between robust poisson models and log-binomial models when estimating relative risks for common binary outcomes: a simulation study. *BMC Med Res Methodol* 2014;**14**:82. doi:10.1186/1471-2288-14-82

For peer review only - http://bmjopen.bmj.com/site/about/guidelines.xhtml

29 Quaiyum MA, Gazi R, Khan AI, *et al.* Programmatic aspects of dropouts in child vaccination in Bangladesh: findings from a prospective study. *Asia Pac J Public Health* 2011;**23**:141–50. doi:10.1177/1010539509342119

30 Hussain A, Ali SM, Kvåle G. Determinants of mortality among children in the urban slums of Dhaka city, Bangladesh. *Trop Med Int Health* 1999;**4**:758–64.

31 Hidalgo B, Goodman M. Multivariate or Multivariable Regression? *Am J Public Health* 2012;**103**:39–40. doi:10.2105/AJPH.2012.300897

32 Restrepo-Méndez MC, Barros AJ, Wong KL, *et al.* Inequalities in full immunization coverage: trends in low- and middle-income countries. *Bull World Health Organ* 2016;**94**:794–805B. doi:http://dx.doi.org/10.2471/BLT.15.162172

33 Fox J, Weisberg S. *An R Companion to Applied Regression. Chapter 6: Diagnosing Problems in Linear and Generalized Linear Models.* SAGE Publications 2011. https://us.sagepub.com/sites/default/files/upm-binaries/38503_Chapter6.pdf

34 Heller LR. Do slums matter? Location and early childhood preventive care choices among urban residents of Bangladesh. *Soc Sci Med* 2013;**94**. doi:10.1016/j.socscimed.2013.06.011

35 Miles M, Ryman TK, Dietz V, *et al.* Validity of vaccination cards and parental recall to estimate vaccination coverage: a systematic review of the literature. *Vaccine* 2013;**31**:1560–8. doi:10.1016/j.vaccine.2012.10.089

36 Murray CJ, Shengelia B, Gupta N, *et al.* Validity of reported vaccination coverage in 45 countries. *Lancet* 2003;**362**:1022–7.

37 Luman ET, Ryman TK, Sablan M. Estimating vaccination coverage: validity of household-retained vaccination cards and parental recall. *Vaccine* 2009;**27**:2534–9. doi:10.1016/j.vaccine.2008.10.002

38 Brown J, Monasch R, Bicego G, *et al. An assessment of the quality of national child immunization coverage estimates in population-based surveys*. MEASURE Evaluation, Carolina Population Center, University of North Carolina at Chapel Hill 2002.

39 Khan A, Khan A. Validating measures of immunization coverage: lessons from international experience. USAID 2012. http://www.resdev.org/files/policy_brief/10/Policy%20brief%2010%20-%20Validation%20of%20Immunization%20Literature.pdf (accessed 2 Oct 2015).

40 Fujita M, Sato Y, Nagashima K, *et al.* Impact of geographic accessibility on utilization of the annual health check-ups by income level in Japan: A multilevel analysis. *PLoS One* 2017;**12**:e0177091. doi:10.1371/journal.pone.0177091

41 Bokade CM, Madhura AD, Bagul AS, *et al.* Predictors of mortality in children due to severe and very severe pneumonia. *Niger Med J* 2015;**56**:287.

42 National Institute of Population Research and Training - NIPORT/Bangladesh, Mitra and Associates, ICF International. *Bangladesh Demographic and Health Survey 2014*. Dhaka, Bangladesh: NIPORT, Mitra and Associates, and ICF International 2016. http://dhsprogram.com/pubs/pdf/FR311/FR311.pdf

For peer review only

43 World Health Organization. *A conceptual framework for action on the social determinants of health*. Geneva: WHO 2010. http://apps.who.int/iris/bitstream/10665/44489/1/9789241500852_eng.pdf (accessed 27 Feb 2017).

44 Christakis DA, Mell L, Wright JA, *et al.* The association between greater continuity of care and timely measles-mumps-rubella vaccination. *Am J Public Health* 2000;**90**:962.

45 Christakis DA, Mell L, Koepsell TD, *et al.* Association of lower continuity of care with greater risk of emergency department use and hospitalization in children. *Pediatrics* 2001;**107**:524–9.

46 Van Walraven C, Oake N, Jennings A, *et al.* The association between continuity of care and outcomes: a systematic and critical review. *J Eval Clin Pract* 2010;**16**:947–56.

47 Kuhn R. Identities in motion: Social exchange networks and rural- urban migration in Bangladesh. *Contributions to Indian Sociology* 2003;**37**:311–37. doi:10.1177/006996670303700113

48 Mills E, Jadad AR, Ross C, *et al.* Systematic review of qualitative studies exploring parental beliefs and attitudes toward childhood vaccination identifies common barriers to vaccination. *J Clin Epidemiol* 11;**58**:1081–8. doi:10.1016/j.jclinepi.2005.09.002

49 Rainey JJ, Watkins M, Ryman TK, *et al.* Reasons related to non-vaccination and under-vaccination of children in low and middle income countries: findings from a systematic review of the published literature, 1999–2009. *Vaccine* 2011;**29**:8215–21.

50 Favin M, Steinglass R, Fields R, *et al.* Why children are not vaccinated: a review of the grey literature. *International Health* 2012;**4**:229–38. doi:10.1016/j.inhe.2012.07.004

51 Sharma V, Singh A, Sharma V. Provider's and User's Perspective about Immunization Coverage among Migratory and Non-migratory Population in Slums and Construction Sites of Chandigarh. *J Urban Health* 2015;**92**:304–12. doi:10.1007/s11524-015-9939-2

For peer review only

For peer review only - http://bmjopen.bmj.com/site/about/guidelines.xhtml

Figures and Tables

Table 1: Mobility status of study households with children age 0-59 months and association with demographic, socioeconomic, and health characteristics using t- and χ^2-tests

	Residentially stable, ≥24 months n=5513		Recently relocated, ≤12 months n=4507		p-value
Demographics	mean	SE	mean	SE	
Number of household members	5.40	0.09	4.60	0.10	**<0.001**
Number of children <5yrs in household	1.28	0.01	1.25	0.01	0.194
Age of index child in months	30.0	0.23	28.7	0.25	**<0.001**
	n	%	n	%	
Sex of index child: Male	2478	45	2048	45	0.622
Socioeconomics					
Mother's education					**<0.001**
No education	1133	21	1162	26	
Some schooling	1142	21	1176	26	
Finished secondary	1849	34	1483	33	
> Secondary	1389	25	686	15	
Father's education					**<0.001**
No education	1261	23	1139	25	
Some schooling	913	17	911	20	
Finished secondary	1525	28	1372	30	
> Secondary	1814	33	1085	24	
Occupation of household head					**<0.001**
Unemployed or other	482	9	232	5	
Daily labor	916	17	1218	27	
Shopkeeper or merchant	1787	32	1058	23	
Salaried service	2328	42	1999	44	
Monthly household income[a]					**<0.001**
≤ 5,000 taka (US $73)	971	18	1080	24	

For peer review only

5,001 – 10,000 taka	1634	30	1925	43	
> 10,000 taka (US $145)	2900	53	1498	33	
Household wealth index[b]					**<0.001**
Poorest	1092	20	1110	25	
Lower middle	922	17	1021	23	
Middle	966f	18	911	20	
Upper middle	1455	26	1155	26	
Richest	1078	20	310	7	
Health services knowledge					
Knowledge of local hospital	4709	85	3428	76	**<0.001**
Health outcomes					
Severe acute respiratory illness suffered by index child within 12 months[c]	763	14	695	15	**0.026**
Meningitis/encephalitis suffered by index child within 12 months[d]	185	3	164	4	0.443

[a]12 respondents (8 residentially stable and 4 recently relocated) did not know or did not disclose household income.
[b]Polychoric Principal Components Analysis was used to create a household wealth index including structural housing characteristics, cooking fuel, drinking water, and sanitation.
[c]One residentially stable respondent did not know if child recently had a severe acute respiratory illness.
[d]Three respondents did not know if child recently had a serious illness with mental status changes (2 residentially stable and 1 recently relocated).

For peer review only

For peer review only - http://bmjopen.bmj.com/site/about/guidelines.xhtml

Table 2: *Using vaccination card plus maternal recall*, vaccination coverage among children age 9-59 months and association with mobility status using univariate and multivariable models with modified Poisson regression

	Partial vaccination n=1465 (17%)		Full vaccination[a] n=7043 (83%)		PR[b]	95% CI	p-value
Univariate analyses							
Mobility status	n	%	n	%			
Residentially stable ≥24 months	706	15	4007	85	Reference		
Recently relocated ≤12 months	759	20	3036	80	**0.94**	**0.91-0.97**	**<0.001**
Demographics	mean	SE	mean	SE			
Number of children <5yrs in household	1.31	0.02	1.25	0.01	**0.96**	**0.93-0.98**	**0.002**
Age of index child in months	32.0	0.44	34.2	0.16	**1.002**	**1.001-1.003**	**<0.001**
Socioeconomics							
Mother's education	n	%	n	%			
No education	629	31	1377	69	Reference		
Some schooling	401	21	1540	79	**1.16**	**1.10-1.21**	**<0.001**
Finished secondary	323	11	2513	89	**1.29**	**1.23-1.36**	**<0.001**
> Higher secondary	112	6	1613	94	**1.36**	**1.30-1.43**	**<0.001**
Occupation of household head							
Unemployed or other	106	17	505	83	Reference		
Daily labor	524	29	1283	71	**0.86**	**0.82-0.90**	**<0.001**
Shopkeeper or merchant	348	14	2084	86	1.04	0.99-1.08	0.112
Salaried service	487	13	3171	87	**1.05**	**1.01-1.09**	**0.029**
Household wealth status (PCA[c])							
Poorest	596	32	1290	68	Reference		
Lower middle	334	20	1298	80	**1.16**	**1.09-1.24**	**<0.001**
Middle	242	15	1348	85	**1.24**	**1.16-1.32**	**<0.001**
Upper middle	193	9	2043	91	**1.34**	**1.25-1.42**	**<0.001**
Richest	100	9	1064	91	**1.34**	**1.25-1.43**	**<0.001**
Health services knowledge							
Does *not* have knowledge of local hospital	354	23	1218	77	Reference		
Has knowledge of local hospital	1111	16	5825	84	**1.08**	**1.05-1.12**	**<0.001**

For peer review only - http://bmjopen.bmj.com/site/about/guidelines.xhtml

Multivariable analyses with different models			
Mobility, adjusting for demographics (# of children and age of index child)	**0.94**	**0.92-0.97**	**<0.001**
Mobility, adjusting for socioeconomics (education, occupation, and wealth)	**0.97**	**0.95-0.99**	**0.009**
Mobility, adjusting for demographics and socioeconomics	**0.97**	**0.95-0.99**	**0.016**

[a]Full vaccination coverage per Expanded Programme on Immunization includes 1 dose of BCG, 3 doses of polio, 3 doses of DPT, and 1 dose of measles vaccines.
[b]PR, Prevalence Ratio.
[c]Polychoric Principal Components Analysis was used to create a household wealth index including structural housing characteristics, cooking fuel, drinking water, and sanitation.

For peer review only

For peer review only - http://bmjopen.bmj.com/site/about/guidelines.xhtml

Table 3: ***Using vaccination card only*****, vaccination coverage among children age 9-59 months who have vaccination cards and association with mobility status using univariate and multivariable models with modified Poisson regression**

	Partial vaccination n=564 (15%)		Full vaccination[a] n=3085 (85%)		PR[b]	95% CI	p-value
Univariate analyses							
Mobility status	n	%	n	%			
Residentially stable ≥24 months	329	14	1948	86	Reference		
Recently relocated ≤12 months	235	17	1137	83	0.97	0.93-1.00	0.083
Demographics	mean	SE	mean	SE			
Number of children <5yrs in household	1.26	0.02	1.24	0.01	0.99	0.96-1.02	0.486
Age of index child in months	27.1	0.64	29.6	0.25	**1.002**	**1.001-1.003**	**0.001**
Socioeconomics							
Mother's education	n	%	n	%			
No education	165	28	430	72	Reference		
Some schooling	123	16	654	84	**1.16**	**1.09-1.24**	**<0.001**
Finished secondary	186	14	1158	86	**1.19**	**1.11-1.28**	**<0.001**
> Higher secondary	90	10	843	90	**1.25**	**1.17-1.33**	**<0.001**
Occupation of household head							
Unemployed or other	35	13	238	87	Reference		
Daily labor	148	26	413	74	**0.84**	**0.78-0.91**	**<0.001**
Shopkeeper or merchant	143	13	921	87	0.99	0.94-1.04	0.778
Salaried service	238	14	1513	86	0.99	0.95-1.04	0.704
Household wealth status (PCA[c])							
Poorest	148	27	401	73	Reference		
Lower middle	131	21	503	79	**1.09**	**1.02-1.16**	**0.009**
Middle	97	15	572	86	**1.17**	**1.09-1.26**	**<0.001**
Upper middle	110	10	1005	90	**1.23**	**1.15-1.32**	**<0.001**
Richest	78	11	604	89	**1.21**	**1.13-1.30**	**<0.001**
Health services knowledge							
Does *not* have knowledge of local hospital	101	19	434	81	Reference		
Has knowledge of local hospital	463	15	2651	85	**1.05**	**1.01-1.09**	**0.025**

For peer review only - http://bmjopen.bmj.com/site/about/guidelines.xhtml

For peer review only

Multivariable analyses with different models				
Mobility, adjusting for demographics (# of children and age of index child)		0.97	0.94-1.01	0.126
Mobility, adjusting for socioeconomics (education, occupation, and wealth)		0.98	0.95-1.02	0.308
Mobility, adjusting for demographics and socioeconomics		0.98	0.95-1.02	0.396

[a]Full vaccination coverage per Expanded Programme on Immunization includes 1 dose of BCG, 3 doses of polio, 3 doses of DPT, and 1 dose of measles vaccines.
[b]PR, Prevalence Ratio.
[c]Polychoric Principal Components Analysis was used to create a household wealth index including structural housing characteristics, cooking fuel, drinking water, and sanitation.

For peer review only

For peer review only - http://bmjopen.bmj.com/site/about/guidelines.xhtml

Table 4: Qualified provider visits for severe acute respiratory illness within prior year among children < 5 years old and association with mobility using univariate and multivariable models with modified Poisson regression

	No qualified provider for severe ARI[a] n=358 (25%)		Qualified provider for severe ARI[a] n=1100 (75%)		PR[b]	95% CI	p-value
Univariate analyses							
Mobility status	n	%	n	%			
Residentially stable ≥24 months	141	18	622	82	Reference		
Recently relocated ≤12 months	217	31	478	69	**0.84**	**0.79-0.90**	**<0.001**
Demographics	mean	SE	mean	SE			
Number of children <5yrs in household	1.23	0.03	1.25	0.02	1.02	0.97-1.08	0.437
Age of index child in months	29.1	0.83	23.7	0.44	**0.994**	**0.992-0.996**	**<0.001**
Socioeconomics							
Mother's education	n	%	n	%			
No education	134	38	220	62	Reference		
Some schooling	127	32	267	68	1.09	0.96-1.23	0.170
Finished secondary	79	16	425	84	**1.36**	**1.20-1.54**	**<0.001**
> Higher secondary	18	9	188	91	**1.47**	**1.30-1.66**	**<0.001**
Occupation of household head							
Unemployed or other	24	23	82	77	Reference		
Daily labor	139	36	244	64	**0.82**	**0.72-0.94**	**0.005**
Shopkeeper or merchant	89	22	320	78	1.01	0.90-1.14	0.848
Salaried service	106	19	454	81	1.05	0.93-1.18	0.435
Household wealth status (PCA[c])							
Poorest	154	40	228	60	Reference		
Lower middle	93	30	220	70	**1.18**	**1.02-1.37**	**0.031**
Middle	59	21	220	79	**1.32**	**1.16-1.51**	**<0.001**
Upper middle	37	12	277	88	**1.48**	**1.31-1.67**	**<0.001**
Richest	15	9	155	91	**1.53**	**1.35-1.73**	**<0.001**
Health services knowledge							
Does *not* have knowledge of local hospital	114	49	118	51	Reference		

For peer review only - http://bmjopen.bmj.com/site/about/guidelines.xhtml

Has knowledge of local hospital	244	20	982	80	**1.57**	**1.34-1.85**	**<0.001**
Multivariable analyses with different models							
Mobility, adjusting for demographics (# of children and age of index child)					**0.84**	**0.79-0.89**	**<0.001**
Mobility, adjusting for socioeconomics (education, occupation, and wealth)					**0.89**	**0.84-0.94**	**<0.001**
Mobility, adjusting for demographics and socioeconomics					**0.88**	**0.84-0.93**	**<0.001**

[a]ARI, Acute Respiratory Illness.
[b]PR, Prevalence Ratio.
[c]Polychoric Principal Components Analysis was used to create a household wealth index including structural housing characteristics, cooking fuel, drinking water, and sanitation.

For peer review only

For peer review only - http://bmjopen.bmj.com/site/about/guidelines.xhtml

Figure 1: Children sampled by mobility status for healthcare utilization and vaccination coverage analysis
Note: *ARI = Acute Respiratory Illness

For peer review only

For peer review only - http://bmjopen.bmj.com/site/about/guidelines.xhtml

Supplemental Figure 1: Cook's distance calculations to examine influential observations

Cook's distance plots of multivariable models examining mobility, demographics, and socioeconomics for: vaccination using card plus maternal recall among children 9-59 months (A), vaccination using card only among children 9-59 months (B), qualified provider visits for respiratory infections among children <5 years old (C), vaccination using card plus maternal recall among children 9-23 months (D), and vaccination using card only among children 9-23 months (E). The red lines indicate threshold value of 4/n where n=number of observations.

For peer review only

For peer review only - http://bmjopen.bmj.com/site/about/guidelines.xhtml

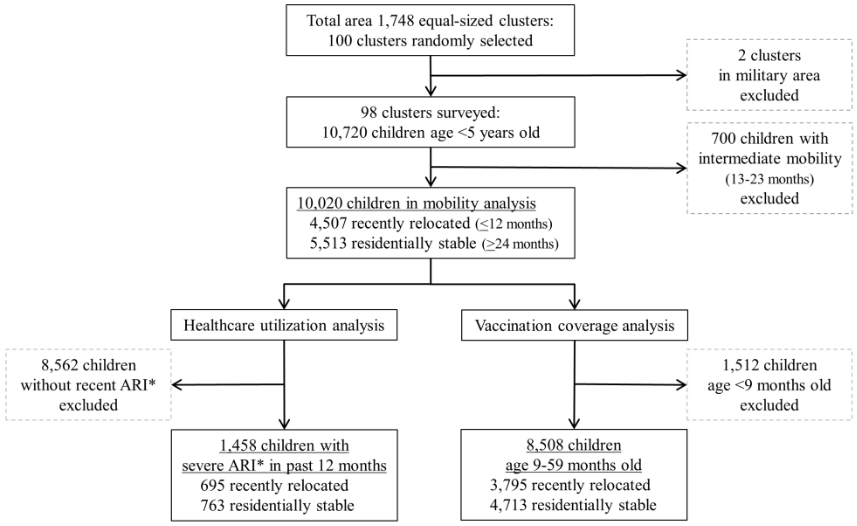

Figure 1: Children sampled by mobility status for healthcare utilization and vaccination coverage analysis
Note: *ARI = Acute Respiratory Illness

Figure 1: Children sampled by mobility status for healthcare utilization and vaccination coverage analysis
Note: *ARI = Acute Respiratory Illness

254x190mm (300 x 300 DPI)

Effect of household relocation on child vaccination and health service utilization in Dhaka, Bangladesh: a cross-sectional community survey

SUPPLEMENTAL FIGURES AND TABLES

Supplemental Table 1: Household wealth principal components analysis coding and variable loading

Indicator	Coding	Loading
Number of rooms	Continuous	0.337
Housing arrangement	1 = Free<br>2 = Rental<br>3 = Owned	0.235
Roof	1 = Natural roof (bamboo/thatch)<br>2 = Rudimentary roof (tin)<br>3 = Finished roof (cement/concrete/tiled)<br>4 = Other	0.392
Walls	1 = Natural walls (jute/bamboo/mud)<br>2 = Rudimentary walls (wood)<br>3 = Finished walls (tin/brick/cement)<br>4 = Other	0.316
Floor	1 = Natural floor (earth/bamboo)<br>2 = Rudimentary floor (wood)<br>3 = Finished floor (cement/concrete)<br>4 = Other	0.377
Cooking fuel	1 = Natural (wood/grass/dung)<br>2 = Coal/charcoal<br>3 = Kerosene<br>4 = Electricity<br>5 = Gas (liquid/biogas)<br>6 = Other	0.350
Drinking water	1 = Unimproved (unprotected/surface/bottled)<br>2 = Improved but not piped<br>3 = Improved and piped into yard or private dwelling<br>4 = Other	0.170
Sanitation	1 = Unimproved (open defecation/hanging/open or broken pit)<br>2 = Improved but not piped	0.374

For peer review only - http://bmjopen.bmj.com/site/about/guidelines.xhtml

	3 = Improved and piped sewer<br>4 = Other	
Mobile phone ownership	0 = No<br>1 = Yes	0.380

Note: 51% of overall variance was explained by the first component.

For peer review only

For peer review only - http://bmjopen.bmj.com/site/about/guidelines.xhtml

Supplemental Table 2: *Using vaccination card plus maternal recall*, vaccination coverage among children age 9-23 months and association with mobility status using univariate and multivariable models with modified Poisson regression

	Partial vaccination n=518 (21%)		Full vaccination[a] n=1906 (79%)		PR[b]	95% CI	p-value
Univariate analyses							
Mobility status	n	%	n	%			
Residentially stable ≥24 months	243	19	1053	81	Reference		
Recently relocated ≤12 months	275	24	853	76	**0.93**	**0.88-0.98**	**0.009**
Demographics	mean	SE	mean	SE			
Number of children <5yrs in household	1.31	0.03	1.24	0.01	**0.94**	**0.89-0.99**	**0.035**
Age of index child in months	14.73	0.20	16.35	0.11	**1.02**	**1.02-1.03**	**<0.001**
Socioeconomics							
Mother's education	n	%	n	%			
No education	207	41	303	59	Reference		
Some schooling	121	22	433	78	**1.32**	**1.18-1.47**	**<0.001**
Finished secondary	141	16	719	84	**1.41**	**1.27-1.56**	**<0.001**
> Higher secondary	49	10	451	90	**1.52**	**1.36-1.69**	**<0.001**
Occupation of household head							
Unemployed or other	26	15	147	85	Reference		
Daily labor	195	38	318	62	**0.73**	**0.66-0.81**	**<0.001**
Shopkeeper or merchant	124	19	538	81	0.96	0.88-1.04	0.287
Salaried service	173	16	903	84	0.99	0.91-1.07	0.752
Household wealth status (PCA[c])							
Poorest	212	40	321	60	Reference		
Lower middle	114	25	348	75	**1.25**	**1.11-1.40**	**<0.001**
Middle	84	18	382	82	**1.36**	**1.20-1.54**	**<0.001**
Upper middle	59	10	560	90	**1.50**	**1.34-1.69**	**<0.001**
Richest	49	14	295	86	**1.42**	**1.26-1.61**	**<0.001**
Health services knowledge							
Does *not* have knowledge of local hospital	127	28	324	72	Reference		
Has knowledge of local hospital	391	20	1582	80	**1.12**	**1.05-1.19**	**<0.001**

For peer review only

For peer review only - http://bmjopen.bmj.com/site/about/guidelines.xhtml

Multivariable analyses with different models				
Mobility, adjusting for demographics (# of children and age of index child)		**0.93**	**0.88-0.98**	**0.007**
Mobility, adjusting for socioeconomics (education, occupation, and wealth)		0.96	0.91-1.01	0.136
Mobility, adjusting for demographics and socioeconomics		0.96	0.92-1.01	0.138

[a]Full vaccination coverage per Expanded Programme on Immunization includes 1 dose of BCG, 3 doses of polio, 3 doses of DPT, and 1 dose of measles vaccines.
[b]PR, Prevalence Ratio.
[c]Polychoric Principal Components Analysis was used to create a household wealth index including structural housing characteristics, cooking fuel, drinking water, and sanitation.

For peer review only

Supplemental Table 3: *Using vaccination card only*, vaccination coverage among children age 9-23 months who have vaccination cards and association with mobility status using univariate and multivariable models with modified Poisson regression

	Partial vaccination n=281 (19%)		Full vaccination[a] n=1222 (81%)		PR[b]	95% CI	p-value
Univariate analyses							
Mobility status	n	%	n	%			
Residentially stable ≥24 months	156	18	715	82	Reference		
Recently relocated ≤12 months	125	20	507	80	0.97	0.92-1.04	0.469
Demographics	mean	SE	mean	SE			
Number of children <5yrs in household	1.23	0.03	1.21	0.01	0.98	0.93-1.04	0.543
Age of index child in months	14.4	0.27	15.9	0.15	**1.02**	**1.01-1.03**	**<0.001**
Socioeconomics							
Mother's education	n	%	n	%			
No education	94	35	176	65	Reference		
Some schooling	57	17	286	83	**1.28**	**1.15-1.42**	**<0.001**
Finished secondary	93	17	461	83	**1.28**	**1.14-1.43**	**<0.001**
> Higher secondary	37	11	299	89	**1.37**	**1.22-1.52**	**<0.001**
Occupation of household head							
Unemployed or other	14	12	100	88	Reference		
Daily labor	89	33	184	67	**0.77**	**0.68-0.87**	**<0.001**
Shopkeeper or merchant	66	16	352	84	0.96	0.88-1.04	0.327
Salaried service	112	16	586	84	0.96	0.88-1.04	0.297
Household wealth status (PCA[c])							
Poorest	84	32	179	68	Reference		
Lower middle	67	24	211	76	1.12	0.99-1.25	0.062
Middle	52	17	248	83	**1.21**	**1.08-1.37**	**0.002**
Upper middle	42	10	381	90	**1.32**	**1.18-1.48**	**<0.001**
Richest	36	15	203	85	**1.25**	**1.10-1.41**	**<0.001**
Health services knowledge							
Does *not* have knowledge of local hospital	55	22	194	78	Reference		
Has knowledge of local hospital	226	18	1028	82	1.05	0.98-1.13	0.141

For peer review only

For peer review only - http://bmjopen.bmj.com/site/about/guidelines.xhtml

Multivariable analyses with different models				
Mobility, adjusting for demographics (# of children and age of index child)		0.98	0.93-1.04	0.582
Mobility, adjusting for socioeconomics (education, occupation, and wealth)		1.00	0.94-1.06	0.948
Mobility, adjusting for demographics and socioeconomics		1.00	0.95-1.07	0.888

[a]Full vaccination coverage per Expanded Programme on Immunization includes 1 dose of BCG, 3 doses of polio, 3 doses of DPT, and 1 dose of measles vaccines.
[b]PR, Prevalence Ratio.
[c]Polychoric Principal Components Analysis was used to create a household wealth index including structural housing characteristics, cooking fuel, drinking water, and sanitation.

For peer review only

For peer review only - http://bmjopen.bmj.com/site/about/guidelines.xhtml

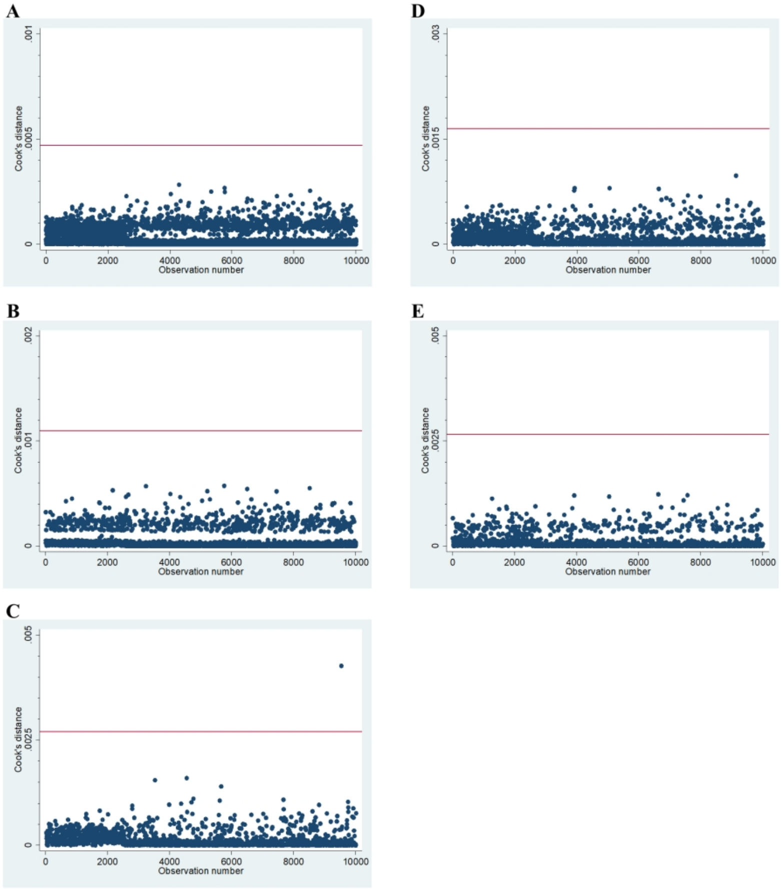

Supplemental Figure 1: Cook's distance calculations to examine influential observations
Cook's distance plots of multivariable models examining mobility, demographics, and socioeconomics for: vaccination using card plus maternal recall among children 9-59 months (A), vaccination using card only among children 9-59 months (B), qualified provider visits for respiratory infections among children <5 years old (C), vaccination using card plus maternal recall among children 9-23 months (D), and vaccination using card only among children 9-23 months (E). The red lines indicate threshold value of 4/n where n=number of observations.

190x254mm (300 x 300 DPI)

Effect of household relocation on child vaccination and health service utilization in Dhaka, Bangladesh: a cross-sectional community survey

STROBE 2007 (v4) Statement—Checklist of items that should be included in reports of *cross-sectional studies*

Section/Topic	Item #	Recommendation	Reported on page #
**Title and abstract**	1	(*a*) Indicate the study's design with a commonly used term in the title or the abstract	1
		(*b*) Provide in the abstract an informative and balanced summary of what was done and what was found	2
**Introduction**			
Background/rationale	2	Explain the scientific background and rationale for the investigation being reported	4
Objectives	3	State specific objectives, including any prespecified hypotheses	6
**Methods**			
Study design	4	Present key elements of study design early in the paper	6
Setting	5	Describe the setting, locations, and relevant dates, including periods of recruitment, exposure, follow-up, and data collection	6
Participants	6	(*a*) Give the eligibility criteria, and the sources and methods of selection of participants	7
Variables	7	Clearly define all outcomes, exposures, predictors, potential confounders, and effect modifiers. Give diagnostic criteria, if applicable	7
Data sources/ measurement	8*	For each variable of interest, give sources of data and details of methods of assessment (measurement). Describe comparability of assessment methods if there is more than one group	7
Bias	9	Describe any efforts to address potential sources of bias	8
Study size	10	Explain how the study size was arrived at	7
Quantitative variables	11	Explain how quantitative variables were handled in the analyses. If applicable, describe which groupings were chosen and why	8
Statistical methods	12	(*a*) Describe all statistical methods, including those used to control for confounding	8
		(*b*) Describe any methods used to examine subgroups and interactions	8
		(*c*) Explain how missing data were addressed	8
		(*d*) If applicable, describe analytical methods taking account of sampling strategy	8

For peer review only

		(*e*) Describe any sensitivity analyses	8
**Results**			
Participants	13*	(a) Report numbers of individuals at each stage of study—eg numbers potentially eligible, examined for eligibility, confirmed eligible, included in the study, completing follow-up, and analysed	9
		(b) Give reasons for non-participation at each stage	9
		(c) Consider use of a flow diagram	Figure 1
Descriptive data	14*	(a) Give characteristics of study participants (eg demographic, clinical, social) and information on exposures and potential confounders	9
		(b) Indicate number of participants with missing data for each variable of interest	9
Outcome data	15*	Report numbers of outcome events or summary measures	9
Main results	16	(*a*) Give unadjusted estimates and, if applicable, confounder-adjusted estimates and their precision (eg, 95% confidence interval). Make clear which confounders were adjusted for and why they were included	10
		(*b*) Report category boundaries when continuous variables were categorized	9
		(*c*) If relevant, consider translating estimates of relative risk into absolute risk for a meaningful time period	n/a
Other analyses	17	Report other analyses done—eg analyses of subgroups and interactions, and sensitivity analyses	10
**Discussion**			
Key results	18	Summarise key results with reference to study objectives	11
Limitations	19	Discuss limitations of the study, taking into account sources of potential bias or imprecision. Discuss both direction and magnitude of any potential bias	13
Interpretation	20	Give a cautious overall interpretation of results considering objectives, limitations, multiplicity of analyses, results from similar studies, and other relevant evidence	15
Generalisability	21	Discuss the generalisability (external validity) of the study results	14
**Other information**			
Funding	22	Give the source of funding and the role of the funders for the present study and, if applicable, for the original study on which the present article is based	17

*Give information separately for cases and controls in case-control studies and, if applicable, for exposed and unexposed groups in cohort and cross-sectional studies.

Note: An Explanation and Elaboration article discusses each checklist item and gives methodological background and published examples of transparent reporting. The STROBE checklist is best used in conjunction with this article (freely available on the Web sites of PLoS Medicine at http://www.plosmedicine.org/, Annals of Internal Medicine at http://www.annals.org/, and Epidemiology at http://www.epidem.com/). Information on the STROBE Initiative is available at www.strobe-statement.org.

For peer review only

BMJ Open

Effect of household relocation on child vaccination and health service utilization in Dhaka, Bangladesh: a cross-sectional community survey

Journal:	*BMJ Open*
Manuscript ID	bmjopen-2018-026176.R2
Article Type:	Research
Date Submitted by the Author:	08-Feb-2019
Complete List of Authors:	Horng, Lily; Stanford University, Infectious Diseases and Geographic Medicine<br>Kakoly, Nadira Sultana; International Centre for Diarrheal Disease Research, Bangladesh (icddr, b)<br>Abedin, Jaynal ; International Centre for Diarrheal Disease Research, Bangladesh (icddr, b)<br>Luby, Stephen; Stanford University, Infectious Diseases and Geographic Medicine; International Centre for Diarrheal Disease Research, Bangladesh (icddr, b)
**Primary Subject Heading**:	Global health
Secondary Subject Heading:	Epidemiology, Paediatrics, Public health, Infectious diseases
Keywords:	Community child health < PAEDIATRICS, PUBLIC HEALTH, Public health < INFECTIOUS DISEASES, Paediatric infectious disease & immunisation < PAEDIATRICS, EPIDEMIOLOGY

SCHOLARONE™
Manuscripts

Effect of household relocation on child vaccination and health service utilization in Dhaka, Bangladesh: a cross-sectional community survey

Lily M. Horng[1]*, Nadira K. Sultana[2], Jaynal Abedin[2], Stephen P. Luby[1, 2]

1 Division of Infectious Diseases & Geographic Medicine, Department of Medicine, Stanford University, Stanford, California, 94305, USA

2 International Centre for Diarrheal Disease Research, Bangladesh (icddr, b), Dhaka 1000, Bangladesh

*Corresponding author:

Lily M. Horng, MD MPH

Division of Infectious Diseases and Geographic Medicine,

Stanford University, Stanford, California, 94305, USA

+16507212730

lhorng@stanford.edu

Word count (excluding title page, abstract, references, figures and tables): 4,303 words

For peer review only

For peer review only - http://bmjopen.bmj.com/site/about/guidelines.xhtml

Abstract

Objective To explore the relationship between household relocation and use of vaccination and health services for severe acute respiratory illness (ARI) among children in Dhaka, Bangladesh.

Design Analysis of cross-sectional community survey data from a prior study examining the impact of *Haemophilus influenzae* type b vaccine introduction in 2009 on meningitis incidence in Bangladesh.

Setting Communities surrounding two large pediatric hospitals in Dhaka, Bangladesh.

Participants Households with children under 5 years old who either recently relocated ≤ 12 months or who were residentially stable living ≥ 24 months in their current residence (total n = 10,020) were selected for this study.

Primary outcome measures Full vaccination coverage among 9-59 month old children and visits to a qualified medical provider for severe ARI among children under 5 years old.

Results Using vaccination cards with maternal recall, full vaccination was 80% among recently relocated children (n=3,795) and 85% among residentially stable children (n=4,713; χ^2=37.2, p<0.001). Among children with ARI in the prior year, 69% of recently relocated children (n=695) had visited a qualified provider compared to 82% of residentially stable children (n=763; χ^2=31.9, p<0.001). After adjusting for demographic and socioeconomic characteristics, recently relocated children were less likely to be fully vaccinated (prevalence ratio [PR] 0.97; 95% confidence interval [CI] 0.95-0.99; p=0.016) and to have visited a qualified provider for ARI (PR 0.88; 95% CI 0.84-0.93; p<0.001).

Conclusions Children in recently relocated households in Dhaka, Bangladesh have decreased use of vaccination and qualified health services for severe acute respiratory illnesses.

Strengths and limitations of this study

For peer review only - http://bmjopen.bmj.com/site/about/guidelines.xhtml

- This study examined a rich dataset from prior community surveys in Dhaka, Bangladesh to explore associations between household relocation and utilization of vaccination and qualified child health services.
- Vaccination was evaluated using different measurements and age ranges to explore trends in the relationship between mobility and vaccination.
- Effect of household relocation on use of child health services was found even after adjusting for socioeconomic factors known to impact health-seeking behavior.
- Limitations include lack of detailed data on mobility patterns and costs of health services.

For peer review only - http://bmjopen.bmj.com/site/about/guidelines.xhtml

Introduction

Pneumonia or acute respiratory illness (ARI) is the leading cause of death globally in children under 5 years old, and lower respiratory tract infections caused an estimated 652,572 child deaths in 2016.[1] Many causes of ARI are preventable by vaccines such as *Streptococcus pneumoniae* (attributed to 52% of global pneumonia child deaths) and *Haemophilus influenzae* type b (Hib) (7% of deaths).[1] The majority of the world's population now lives in urban areas, and this population is expected to grow from 54% in 2014 to 66% in 2050, an estimated increase of 2.5 billion people.[2,3] With this increase, 90% of growth is projected in Asia and Africa.[3] The population living in urban slums is also expected to increase from 881 million in 2014 to 2 billion in 2030, in large part due to rural-urban migration.[2,4]

In many low- and middle-income countries, vaccination and childhood mortality rates among urban poor are worse than among other urban groups and even rural populations.[5,6] In addition, residents of slums have poor health outcomes due to lack of reliable access to housing, clean water, sanitation, education, and health services.[4,5,7–9] In Nigeria, a 2010 study examining 2003 Demographic and Health Survey data of 6,029 children 12 months and older found full immunization among 24.3% of rural non-migrant, 15.2% of urban non-migrant, and 8.5% of rural-urban migrant children.[10] In Bangladesh, a comparison study of the 2013 Urban Health Survey and 2014 Demographic and Health Survey found under-5 child mortality rates of 46 per 1,000 livebirths nationally, 41 in Dhaka, 49 in rural areas, and 57 in urban slums.[5] One recent systematic review found community factors associated with vaccination coverage in the urban poor included socio-economic characteristics, vaccination knowledge and beliefs, access to care, and recent rural-urban migration.[6]

Residential mobility has been recognized as an important contributor to healthcare use in high-income countries, with relocation associated with decreased use of preventive and curative services.[11,12] One study using a 1998 United States national health survey found that duration, distance, and frequency of moving were all predictors of decreased use of child

For peer review only

For peer review only - http://bmjopen.bmj.com/site/about/guidelines.xhtml

health services even after accounting for sociodemographic factors. Households who had moved within 12 months accessed fewer preventive child health services compared to households living in their current residence over 36 months (odds ratio 3.1, 95% confidence interval [CI] 2.5-3.7).[12] Recently relocated households also accessed fewer curative services (odds ratio 3.3, 95% CI 2.6-4.2).[12] Frequent moving also impacted children's long-term cognitive function and behavioral problems into adulthood.[13] Moving can impact health through various social determinants. In one conceptual framework by the World Health Organization (WHO), structural determinants of social, economic, and political contexts influence intermediary determinants of material, behavioral, psychosocial, and health system factors that ultimately shape an individual's health.[14] Relocation can improve an individual's socioeconomic position in the long term, but mobility often disrupts material resources, psychosocial support, and health care access.

Few studies examine mobility and healthcare utilization in low- and middle-income countries despite high population relevance: approximately 43% of urban residents in middle-income countries and 78% in low-income countries live in slums.[3,4,6,10,15,16] In the 2010 Nigeria study, urban non-migrant children had 1.7 times higher odds of being fully immunized than rural-urban migrants (univariate odds ratio [OR] 1.67, 95% confidence interval [CI] 1.20-2.32).[10] This association between migration and immunization was independent of demographic factors, but was attenuated and partially explained by socioeconomic characteristics and maternal healthcare utilization in multivariable analyses.[10] In this Nigeria study, migrant status was defined as moving within 10 years.[10] A 2010 cross-sectional survey in India examined 746 rural-urban migrant mothers with children under 2 years old: 339 were 'recent' migrants who moved to Delhi within 5 years and 407 were 'settled' migrants in Delhi at least 5 years.[16] For age-appropriate children, 81% of settled migrants and 64% of recent migrants were fully immunized per national guidelines.[16] Settled migrant children had 1.9 times higher odds of being fully immunized

For peer review only - http://bmjopen.bmj.com/site/about/guidelines.xhtml

than recent migrants after adjusting for demographics, socioeconomics, and maternal healthcare utilization (adjusted OR 1.93, 95% CI 1.18-3.14).[16]

Studying urban health services in Bangladesh is useful because Bangladesh is the world's most densely populated country that is not a city-state: the population of the capital Dhaka will increase from an estimated 16 to 27 million by 2030.[3,17] Furthermore, the government has a strong national Expanded Programme on Immunization and active health systems research.[17,18] In 2011, full vaccination rates among children age 12-23 months were 80% nationally in Bangladesh, 75% in Dhaka, but only 43-67% in Dhaka slums.[17–21] Prior studies found that household turnover was as high as 50% in one year, comprehensive provider-led vaccination interventions were effective but too expensive to sustain, and street children were very hard to reach with interventions in Dhaka.[19,22,23]

To explore the relationship between residential mobility and healthcare utilization in Dhaka, we used data from a study showing Hib vaccine introduction into Bangladesh's Expanded Programme on Immunization in 2009 dramatically reduced rates of Hib meningitis and purulent meningitis in children.[24] We conducted secondary analysis of the Hib impact study's community survey data to determine whether recently relocated children were: 1) less likely to be fully vaccinated per Expanded Programme on Immunization guidelines and 2) less likely to use qualified health services for severe acute respiratory illness than residentially stable children.

Methods

Study design and setting

Hib conjugate vaccine was introduced into Bangladesh's Expanded Programme on Immunization in 2009, and the Hib impact study conducted pre and post-vaccine surveillance of meningitis in children under 5 years old using hospital records and community surveys surrounding two large pediatric hospitals in Dhaka: Dhaka Shishu and Shishu Shastya

For peer review only - http://bmjopen.bmj.com/site/about/guidelines.xhtml

Foundation Hospital.[24] Field researchers consecutively enrolled 100 children discharged with a diagnosis of meningitis and/or encephalitis from the two study hospitals, visited households, and recorded household geographical positional system coordinates. The catchment area was defined as the area containing >80% of households with children discharged with meningitis and within one hour of transport to either hospital. Field teams divided the catchment area into 1,748 equal-sized rectangles and randomly selected 100 rectangles as clusters. Teams surveyed each household with a child under 5 years old within 98 clusters. Two clusters were within a military cantonment, thus inaccessible. Households were asked about: 1) routine vaccinations using vaccination cards and maternal recall and 2) healthcare use for children with illnesses in the prior 12 months suggestive of meningoencephalitis defined as: any serious illness with acute onset of fever with either convulsions or unconsciousness or altered mental status.[24] Data were collected one year before (2008) and after (2010) Hib vaccine introduction.

Study population

We used the Hib impact study's pre-vaccine community surveillance data and included children based on mobility status: 1) children living in their current residence ≤ 12 months who we classified as "recently relocated" and 2) children living ≥ 24 months in their current residence who we classified as "residentially stable". This definition of mobility/migration status has been used in prior studies.[6,12] We excluded children living in their current residence 13-23 months who we classified as "intermediately mobile".

Study outcomes

Our two primary outcomes focused on healthcare utilization: 1) full vaccination among 9-59 month old children and 2) visit to a qualified medical provider among children under 5 years old who had severe acute respiratory illness symptoms within the prior 12 months. We defined full vaccination per Bangladesh Expanded Programme on Immunization

For peer review only - http://bmjopen.bmj.com/site/about/guidelines.xhtml

guidelines in 2008 (before Hib vaccine): 1 dose of Bacillus Calmette-Guérin (BCG) vaccine against tuberculosis; 3 doses of combined vaccine against diphtheria, pertussis, and tetanus (DPT); 3 doses of oral polio vaccine (excluding polio vaccine given at birth); and 1 dose of measles vaccine. Government guidelines recommended children to receive all these vaccinations before 9 months of age. Any doses of pentavalent vaccine, which includes diphtheria, pertussis, tetanus, hepatitis B, and Hib, were included in vaccination analyses. We defined severe acute respiratory illness as cough or difficulty breathing plus any danger sign: stridor, chest in-drawing, difficulty drinking/ breastfeeding, vomiting, cyanosis, convulsions, lethargy, or unconsciousness. We defined a qualified medical provider as having a Bachelor of Medicine degree or higher.

Data analysis

We compared sociodemographic and health characteristics between residentially stable and recently relocated households. For continuous variables, we calculated means with standard errors and t-tests adjusting for cluster. For categorical variables, we calculated percentages and χ^2-tests. To construct a wealth index, we used polychoric principal components analysis (PCA) including: housing (number of rooms; free, rental, or owned housing; main material of roof, walls, and floors), cooking fuel, drinking water, sanitation, and mobile phone ownership.[25–27] We then divided households into wealth quintiles. We did not include durable assets such as furniture items because ownership of these goods could be associated with duration of residency.

To examine the magnitude of association between mobility and study outcomes of vaccination and visit to a qualified provider for severe ARI, we used modified Poisson regression with robust cluster variance to estimate prevalence ratios (PRs).[28,29] We chose modified Poisson regression to model prevalence ratios for common binary outcomes because logistic regression is more applicable to rare outcomes and because log-binomial regression models may fail to converge. We conducted univariate analyses to estimate individual effects

For peer review only - http://bmjopen.bmj.com/site/about/guidelines.xhtml

of mobility, demographics, socioeconomics, and health services knowledge (i.e. knowledge of local hospital) on healthcare utilization. Missing data regarding main study outcome of acute respiratory illness were handled through listwise deletion. Given large number of missing vaccination cards, we analyzed vaccination in two ways: 1) using vaccination cards plus maternal recall and 2) using vaccination cards alone. We conducted multivariable analyses examining the association between mobility and healthcare utilization, adjusting for demographics and socioeconomics known to influence health-seeking behavior.[9,16,30–32] Regression diagnostics included checks for influential observations with Cook's distance calculations.

Ethics

The Ethical Review Committee of the International Centre for Diarrhoeal Disease Research, Bangladesh (icddr,b) reviewed and approved the study protocol. Written informed consent was obtained from all participants before taking part in the initial Hib impact study.

Patient and public involvement

No participants were directly involved in development of the research questions and outcomes. No participants were involved in the design or conduct of the study. There are no plans to disseminate the results of the research individually to study participants.

Results

We surveyed a total of 10,720 households with children less than 5 years old: 42% of households had recently relocated within 12 months, 51% were residentially stable living in their current residence over 24 months, and 7% were intermediately mobile (Figure 1). We excluded from subsequent analyses 700 children living in their current residence 13-23 months and classified as intermediately mobile. For the healthcare utilization analysis, 1,458

For peer review only - http://bmjopen.bmj.com/site/about/guidelines.xhtml

children had severe ARI symptoms within the 12 months prior to survey. For the vaccination analysis, 8,508 children were age 9-59 months and thus should have completed all Expanded Programme on Immunization-recommended vaccinations. Household demographics, parental education, occupation, and hospital knowledge were available for all households. Missing data included: income for 12 households, meningitis symptoms for 3 children, respiratory illness symptoms for 1 child, and vaccination cards for 4,859 children age 9-59 months.

Recently relocated families had smaller households, less education, less wealth, and less knowledge of the local hospital compared to residentially stable families (Table 1). Recently relocated families were poorer: 24% earned less than 5,000 Bangladeshi taka (US $73) per month compared to 18% of residentially stable families. For the wealth index analysis, the first principal component accounted for 51% of overall variance, with largest contributions from roof and floor materials, sanitation, and mobile phone ownership (Supplemental Table 1). Among recently relocated families, 48% were in the two lowest wealth quintiles compared to 37% of residentially stable families. Fewer recently relocated caregivers had knowledge of the local hospital, 76%, compared to residentially stable caregivers, 85% (χ^2=142.3, p<0.001). Similar rates of illness in the 12 months prior to survey were reported by all households: 14-15% of children with symptoms of severe ARI and 3-4% with symptoms of meningitis/encephalitis.

Full vaccination coverage measured by vaccination card plus maternal recall was 83% among all children age 9-59 months (Table 2). Full vaccination was 80% among recently relocated children and 85% among residentially stable children (univariate PR 0.94, 95% CI 0.91-0.97, p<0.001). Vaccination was lower in households with more children and younger children. Socioeconomic factors, especially mother's education, had the strongest association with vaccination. In multivariable analyses, recently relocated children were 3% less likely than residentially stable children to be fully vaccinated even after adjusting for demographic and socioeconomic factors (multivariable PR 0.97, 95% CI 0.95-0.99, p=0.016).

For peer review only - http://bmjopen.bmj.com/site/about/guidelines.xhtml

Vaccination was also analyzed using only vaccination cards (Table 3). At time of survey, only 43% of all children had vaccination cards available. Fewer recently relocated children had vaccination cards, 36%, compared to 48% of residentially stable children (χ^2=126.9, p<0.001). Younger children were more likely to have cards than older children: 62% of children 9-23 months old had vaccination cards as compared to 38% of children 24-59 months old (χ^2=505.7, p<0.001). Full vaccination per vaccination card was 83% among recently relocated children and 86% among residentially stable children (univariate PR 0.97, 95% CI 0.93-1.00, p=0.083). The 9-59 month age range for vaccination analysis allowed inclusion of a larger sample size of children vulnerable to vaccine-preventable disease. In contrast, routine vaccination schedules focus on children <2 years old and full vaccination coverage per Expanded Programme on Immunization in Bangladesh and many other countries is defined in children up to 23 months old.[18,33] Narrowing the age range of vaccination analysis to 9-23 months showed similar results although with smaller sample sizes limiting statistical power to detect mobility-vaccination associations (Supplementary Tables 2 and 3). In addition, using a 10-month age minimum to account for potential delay in measles vaccination recommended at 9 months old showed the same results as a 9-month age cutoff (data not shown). Checking for influential observations with Cook's distances identified no outliers in vaccination analyses (Supplemental Figure 1).[34]

Among all children under 5 years old with severe ARI in the past year, 75% visited a qualified medical provider (Table 4). Fewer recently relocated children with severe ARI saw a qualified provider, 69%, as compared to 82% of residentially stable children (univariate PR 0.84, 95% CI 0.79-0.90, p<0.001). Socioeconomic factors, especially household wealth, were strongly associated with qualified provider visits.

Health services knowledge was also strongly associated with acute healthcare visits: 80% of parents who knew about the local hospital sought ARI treatment from a qualified provider as compared to 51% of parents who did *not* have knowledge of the local hospital (univariate PR 1.57, 95% CI 1.34-1.85, p<0.001). After adjusting for demographic and

For peer review only - http://bmjopen.bmj.com/site/about/guidelines.xhtml

socioeconomic factors, recently relocated households were 11% less likely than residentially stable households to visit a qualified medical provider for children with severe ARI (multivariable PR 0.88, 95% CI 0.84-0.93, $p<0.001$). One outlier was identified by Cook's distances in healthcare utilization analyses (Supplemental Figure 1). Excluding this outlier and using robust error variance resulted in similar results to those presented (data not shown).

Discussion

Recently relocated households were less likely to use both acute and preventive child healthcare services in our study in Dhaka, Bangladesh, and these findings support prior literature exploring the effects of mobility on healthcare utilization.[6,10–13,16] Household relocation had a strong association with decreased use of qualified medical services for severe acute respiratory illness. Similarly, household relocation was associated with decreased vaccination rates although this relationship was less robust. Another key finding was that recently relocated parents were less knowledgeable about the local hospital compared to residentially stable parents, and knowledge of the local hospital had as strong an association with acute healthcare visits as some economic factors. Overall, recently relocated children in our study had slightly lower vaccination rates and markedly lower use of acute healthcare services for ARI than residentially stable children.

Study strengths include data focused on urban Bangladesh and exploring vaccination status using different measurements as well as adjusting for socioeconomic factors when examining mobility and health service utilization. Our study used the Hib impact study's rigorous community surveillance data of households living close to tertiary care pediatric hospitals in Dhaka. Dhaka residents have high mobility and many options for healthcare. Unlike in rural areas, physical access to health services is usually *not* a barrier to healthcare use in urban areas. One study found almost all residents in Dhaka lived within 1 kilometer of primary health services.[35] Routine immunizations are provided free by the government of Bangladesh, but acute care services require out of pocket expenditures which can be a barrier

For peer review only

For peer review only - http://bmjopen.bmj.com/site/about/guidelines.xhtml

to access. Our findings on mobility and child health services use in Dhaka could inform health services work in other urban low- and middle-income country contexts.[3,5]

We analyzed vaccination using vaccination card data augmented with maternal recall, vaccination card data only, and several different age ranges. Accurately measuring vaccinations in children is a known difficulty in public health programs and research studies, especially in low- and middle-income countries where vaccination cards are frequently *not* available. Some studies have found poor agreement between parental recall, vaccination cards, and even official health records.[36–38] By contrast, other studies have found good correlation between maternal report and vaccination cards, and maternal recall is routinely used in Demographic and Health Surveys and Multiple Indicator Cluster Surveys.[39,40] Maternal recall can overestimate or underestimate vaccination history based on education, social desirability bias, and vaccine-specific knowledge. Vaccination card retention itself can be affected by parental education, household wealth, age of child, and even household relocation. Our data showed more missing cards among recently relocated households and in older children, thus those groups are subject to more maternal recall bias. Using only vaccination cards or narrower age ranges in our vaccination analyses resulted in smaller sample sizes which limited statistical power, but all analyses showed similar effect estimates of increased mobility associated with decreased vaccination. Moreover, the association between increased household relocation and decreased health services use was still seen even after adjusting for socioeconomic factors known to impact healthcare use.

Study limitations include lack of data on mobility patterns and health services costs. Information on households' prior residences, distances moved, or frequency of moving was not available in our dataset. Households moving from rural Bangladesh to urban Dhaka, moving long distances, or relocating frequently probably have less knowledge and therefore use of locally available health services.[12] Several studies show that recent rural to urban migration is associated with lower vaccination coverage in children.[6,10,16] Lack of data on mobility patterns in our study precludes evaluation of how magnitude of relocation affected

For peer review only - http://bmjopen.bmj.com/site/about/guidelines.xhtml

healthcare use. Our findings likely underestimate the negative association between mobility and healthcare utilization for households with large migration such as rural to urban migrants as compared to households with intra-Dhaka relocation where one would expect minimal change in health-seeking behavior. We were also unable to examine household relocation timing in relation to healthcare use. Recently relocated households were not asked if healthcare visits occurred before or after moving. Healthcare visits before moving would not be relevant to how mobility affects use of health services after moving. Our findings may underestimate the negative association between mobility and health-seeking behavior because our data included healthcare visits before moving which were unrelated to knowledge of the new geographic area. Timing of healthcare visits in relation to acquiring knowledge of hospital services was also not available, thus there may be reverse causality of recently relocated households gaining knowledge of local providers after seeking care. Ultimately, our results show a modest overall association between mobility and healthcare use which could be elucidated by asking about migration patterns including timing of use of health services.

Our study results may not be as generalizable to populations in urban areas without tertiary care hospitals. Our sampling scheme focused on community catchment areas surrounding tertiary care pediatric hospitals. Advantages of this study design were that it allowed examination of healthcare utilization for severe disease since advanced services were available within a small physical distance. In addition, it was a low-cost way to examine population-level mobility instead of more resource-intensive active surveillance of migrant populations. However, use of health services generally increases with geographic proximity, and studies show this relationship is influenced by many factors including income and slum versus non-slum locations.[35,41] Recently relocated populations may be even more influenced by proximity than residentially stable populations because of fewer socioeconomic resources and lack of knowledge of health services. This would bias our results towards higher rates of health-seeking behavior among recently relocated households. Recently relocated households in areas without tertiary care services may use health services less

For peer review only - http://bmjopen.bmj.com/site/about/guidelines.xhtml

because of transport costs and lack of knowledge of health facilities physically distant. In addition, our study included participants who relocated into the study area, but not people who left the study area. In-migrants and out-migrants may be different in their healthcare utilization patterns, which could also affect generalizability.

Our dataset did not contain cost of services, which is a well-known barrier to healthcare use.[20,21,31] Although vaccinations are provided for free, some non-governmental and private organizations charge fees for patient registration. Even small fees could have lowered vaccination rates. Cost of services, willingness to pay, and underlying finances are strongly linked, thus adjusting for socioeconomic factors of parental education and wealth in our models should have incorporated some cost effects on healthcare use. However, costs could affect recently relocated households disproportionately more than residentially stable households of the same socioeconomic status. One could hypothesize that immediately after relocating, families would first spend money on household goods before preventive medicine fees. Without cost data, we can still conclude from our analysis that increased mobility is associated with decreased healthcare use, but we have limited understanding of mechanisms through which mobility affects healthcare use.

Barriers and delays to using appropriate healthcare services increase mortality.[31,42] One study in India of 290 children hospitalized for pneumonia in a tertiary care center found that delayed hospital referral, defined as three or more days between symptom onset and hospitalization, was associated with increased mortality (OR 52.1, 95% CI 6.7-402.4, $p<0.001$) after adjusting for age, residence in slum, and illness severity.[42] In this study, incomplete immunization was also associated with increased mortality (OR 12.3, 95% CI 2.2-69.9, $p=0.005$).[42] Reasons for delayed care-seeking can include access and cost. While cost does influence healthcare use, parents of sick children usually do seek some treatment. In the 2014 Bangladesh Demographic and Health Survey, 52% of urban parents with children with ARI symptoms in the 2 weeks prior to survey sought treatment from a health facility, 23% went to pharmacies, and 12% went to traditional practitioners.[43] Only 12% of parents with

For peer review only - http://bmjopen.bmj.com/site/about/guidelines.xhtml

sick children sought no health care treatment at all.[43] While cost does not seem a large barrier to seeking any treatment at all, cost likely influences choice of health provider.

Household relocation disrupts prior relationships with healthcare providers and results in lack of familiarity with local services. Studies show that continuity of care is associated with increased vaccination, fewer emergency department visits, and decreased hospitalization among children.[12,44–46] People usually move to new areas because of pre-existing social connections through family, friends, or work.[7,47] These social contacts can act as pathways of important local knowledge, including health services, but recently relocated households have fewer social contacts and access fewer information sources. Other studies have also found that parental attitudes and knowledge are critical factors contributing to use of health services.[6,48–51] One literature review found that *practical* knowledge about vaccination schedule, timing, and logistics had a stronger association with vaccination uptake than scientific knowledge of vaccine names or biologic actions.[50] One study in India of 210 residents in slums and 100 migrant families of construction workers found 28% of slum residents and 64% of migrants identified lack of knowledge of place or time of services as a reason for decreased immunization.[51] Our study did not ask specifically about knowledge of vaccination services, but future research on knowledge of services could help elucidate how to connect new migrants to care.

Our finding that recently relocated children in Dhaka use fewer qualified health services compared to residentially stable children sheds light on health barriers faced by a growing population of children living in urban centers of low- and middle-income countries. Rigorous community surveillance in hospital catchment areas allows for increased understanding of factors affecting access to and use of healthcare services. Policymakers working to improve urban child health could invest in accurate counting of children living in communities with high household turnover in order to connect recently relocated households to already existing local health services. Further studies by researchers on patterns and mechanisms through which mobility affects healthcare use could inform critical intervention

For peer review only - http://bmjopen.bmj.com/site/about/guidelines.xhtml

points. Ultimately, cost-effective and targeted interventions to increase appropriate healthcare use among recently relocated children could improve health of future urban populations.

Contributors

SL was the Principal Investigator and involved in every aspect of the study from conceptualization to data analysis to manuscript writing and submission. NS, JA, and LH were involved in data analysis, and LH drafted the written work. All authors collaborated on and approved the final manuscript.

Competing interests

None declared.

Provenance and peer review

Not commissioned; externally peer reviewed.

Ethical considerations

The Ethical Review Committee of the International Centre for Diarrhoeal Disease Research, Bangladesh (icddr,b) reviewed and approved the study protocol.

Participant consent

Written informed consent was obtained from all participants before taking part in the initial Hib impact study.

Funding

The original Hib impact study data collection was funded by the Bill and Melinda Gates Foundation and the GAVI Hib Initiative (GR-00580). No additional funding was provided for this study.

For peer review only - http://bmjopen.bmj.com/site/about/guidelines.xhtml

Data sharing

Data are available by emailing the corresponding author LH at lhorng@stanford.edu.

Acknowledgements

The authors thank study participants, field researchers, and collaborative partners of the Hib impact study who included Dhaka Shishu microbiology laboratories and the Johns Hopkins Bloomberg School of Public Health. icddr,b is grateful to the Bangladesh, Canada, Sweden and the United Kingdom governments for providing core/unrestricted support.

For peer review only

For peer review only - http://bmjopen.bmj.com/site/about/guidelines.xhtml

References

1 Troeger C, Blacker B, Khalil IA, *et al.* Estimates of the global, regional, and national morbidity, mortality, and aetiologies of lower respiratory infections in 195 countries, 1990–2016: a systematic analysis for the Global Burden of Disease Study 2016. *Lancet Infect Dis* 2018;**18**:1191–210. doi:10.1016/S1473-3099(18)30310-4

2 UN-Habitat. *World Cities Report 2016: Urbanization and Development – Emerging Futures*. New York: UN-Habitat 2016. https://unhabitat.org/books/world-cities-report/ (accessed 12 Jan 2019).

3 United Nations, Department of Economic and Social Affairs, Population Division. World Urbanization Prospects: The 2014 Revision [Highlights]. New York: United Nations 2015. http://esa.un.org/unpd/wup/Publications/Files/WUP2014-Highlights.pdf (accessed 3 Feb 2016).

4 UN-Habitat. *The challenge of slums: global report on human settlements 2003*. New York: UN-Habitat 2003.

5 Ezeh A, Oyebode O, Satterthwaite D, *et al.* The history, geography, and sociology of slums and the health problems of people who live in slums. *The Lancet* 2017;**389**:547–58. doi:10.1016/S0140-6736(16)31650-6

6 Crocker-Buque T, Mindra G, Duncan R, *et al.* Immunization, urbanization and slums – a systematic review of factors and interventions. *BMC Public Health* 2017;**17**:556. doi:10.1186/s12889-017-4473-7

7 Rashid SF. Strategies to reduce exclusion among populations living in urban slum settlements in Bangladesh. *J Health Popul Nutr* 2009;**27**:574–86.

8 UN-Habitat. *State of the World's Cities 2006/7*. New York: : UN-Habitat 2006.

9 Unger A. Children's health in slum settings. *Arch Dis Child* 2013;**98**:799–805. doi:10.1136/archdischild-2011-301621

10 Antai D. Migration and child immunization in Nigeria: individual-and community-level contexts. *BMC Public Health* 2010;**10**:116.

11 Jelleyman T, Spencer N. Residential mobility in childhood and health outcomes: a systematic review. *J Epidemiol Community Health* 2008;**62**:584–92.

12 Fowler MG, Simpson GA, Schoendorf KC. Families on the move and children's health care. *Pediatrics* 1993;**91**:934–40.

13 Wood D, Halfon N, Scarlata D, *et al.* Impact of family relocation on children's growth, development, school function, and behavior. *JAMA* 1993;**270**:1334–8.

14 World Health Organization. *A conceptual framework for action on the social determinants of health*. Geneva: WHO 2010. http://apps.who.int/iris/bitstream/10665/44489/1/9789241500852_eng.pdf (accessed 27 Feb 2017).

15 Awoh AB, Plugge E. Immunisation coverage in rural–urban migrant children in low and middle-income countries (LMICs): a systematic review and meta-analysis. *J Epidemiol Community Health* 2016;**70**:305–11.

For peer review only - http://bmjopen.bmj.com/site/about/guidelines.xhtml

16 Kusuma YS, Kumari R, Pandav CS, *et al.* Migration and immunization: determinants of childhood immunization uptake among socioeconomically disadvantaged migrants in Delhi, India. *Trop Med Int Health* 2010;**15**:1326–32.

17 NIPORT, icddr,b, MEASURE Evaluation. Bangladesh Urban Health Survey 2013 Final Report. 2015. http://www.cpc.unc.edu/measure/resources/publications/tr-15-117 (accessed 3 Feb 2016).

18 Government of Bangladesh. Bangladesh EPI coverage evaluation survey 2011, Expanded Programme on Immunization. 2011. http://www.dghs.gov.bd/images/docs/EPI/CES%20Reports/EPI%20Coverage%20Evaluation%20Survey%202011.pdf

19 Hayford K, Uddin MJ, Koehlmoos TP, *et al.* Cost and sustainability of a successful package of interventions to improve vaccination coverage for children in urban slums of Bangladesh. *Vaccine* 2014;**32**:2294–9. doi:10.1016/j.vaccine.2014.02.075

20 Uddin MJ, Larson CP, Oliveras E, *et al.* Child immunization coverage in urban slums of Bangladesh: impact of an intervention package. *Health Policy Plan* 2010;**25**. doi:10.1093/heapol/czp041

21 Perry H, Weierbach R, Hossain I, *et al.* Childhood immunization coverage in zone 3 of Dhaka City: the challenge of reaching impoverished households in urban Bangladesh. *Bull World Health Organ* 1998;**76**:565–73.

22 Luby SP, Halder AK, Saha SK, *et al.* A low-cost approach to measure the burden of vaccine preventable diseases in urban areas. *Vaccine* 2010;**28**:4903–12. doi:10.1016/j.vaccine.2010.05.040

23 Uddin MJ, Shamsuzzaman M, Horng L, *et al.* Use of mobile phones for improving vaccination coverage among children living in rural hard-to-reach areas and urban streets of Bangladesh. *Vaccine* 2016;**34**:276–83. doi:10.1016/j.vaccine.2015.11.024

24 Sultana NK, Saha SK, Al-Emran HM, *et al.* Impact of introduction of the Haemophilus influenzae type b conjugate vaccine into childhood immunization on meningitis in Bangladeshi infants. *J Pediatr* 2013;**163**:S73–8.

25 Vyas S, Kumaranayake L. Constructing socio-economic status indices: how to use principal components analysis. *Health Policy Plan* 2006;**21**:459–68. doi:10.1093/heapol/czl029

26 Luby SP, Halder AK. Associations among handwashing indicators, wealth, and symptoms of childhood respiratory illness in urban Bangladesh. *Trop Med Int Health* 2008;**13**:835–44. doi:10.1111/j.1365-3156.2008.02074.x

27 Sharker MY, Nasser M, Abedin J, *et al.* The risk of misclassifying subjects within principal component based asset index. *Emerg Themes Epidemiol* 2014;**11**:6.

28 Zou G. A modified poisson regression approach to prospective studies with binary data. *Am J Epidemiol* 2004;**159**:702–6.

29 Chen W, Shi J, Qian L, *et al.* Comparison of robustness to outliers between robust poisson models and log-binomial models when estimating relative risks for common

For peer review only - http://bmjopen.bmj.com/site/about/guidelines.xhtml

binary outcomes: a simulation study. *BMC Med Res Methodol* 2014;**14**:82. doi:10.1186/1471-2288-14-82

30 Quaiyum MA, Gazi R, Khan AI, *et al.* Programmatic aspects of dropouts in child vaccination in Bangladesh: findings from a prospective study. *Asia Pac J Public Health* 2011;**23**:141–50. doi:10.1177/1010539509342119

31 Hussain A, Ali SM, Kvåle G. Determinants of mortality among children in the urban slums of Dhaka city, Bangladesh. *Trop Med Int Health* 1999;**4**:758–64.

32 Hidalgo B, Goodman M. Multivariate or Multivariable Regression? *Am J Public Health* 2012;**103**:39–40. doi:10.2105/AJPH.2012.300897

33 Restrepo-Méndez MC, Barros AJ, Wong KL, *et al.* Inequalities in full immunization coverage: trends in low- and middle-income countries. *Bull World Health Organ* 2016;**94**:794–805B. doi:http://dx.doi.org/10.2471/BLT.15.162172

34 Fox J, Weisberg S. *An R Companion to Applied Regression. Chapter 6: Diagnosing Problems in Linear and Generalized Linear Models.* SAGE Publications 2011. https://us.sagepub.com/sites/default/files/upm-binaries/38503_Chapter6.pdf

35 Heller LR. Do slums matter? Location and early childhood preventive care choices among urban residents of Bangladesh. *Soc Sci Med* 2013;**94**. doi:10.1016/j.socscimed.2013.06.011

36 Miles M, Ryman TK, Dietz V, *et al.* Validity of vaccination cards and parental recall to estimate vaccination coverage: a systematic review of the literature. *Vaccine* 2013;**31**:1560–8. doi:10.1016/j.vaccine.2012.10.089

37 Murray CJ, Shengelia B, Gupta N, *et al.* Validity of reported vaccination coverage in 45 countries. *Lancet* 2003;**362**:1022–7.

38 Luman ET, Ryman TK, Sablan M. Estimating vaccination coverage: validity of household-retained vaccination cards and parental recall. *Vaccine* 2009;**27**:2534–9. doi:10.1016/j.vaccine.2008.10.002

39 Brown J, Monasch R, Bicego G, *et al. An assessment of the quality of national child immunization coverage estimates in population-based surveys*. MEASURE Evaluation, Carolina Population Center, University of North Carolina at Chapel Hill 2002.

40 Khan A, Khan A. Validating measures of immunization coverage: lessons from international experience. USAID 2012. http://www.resdev.org/files/policy_brief/10/Policy%20brief%2010%20-%20Validation%20of%20Immunization%20Literature.pdf (accessed 2 Oct 2015).

41 Fujita M, Sato Y, Nagashima K, *et al.* Impact of geographic accessibility on utilization of the annual health check-ups by income level in Japan: A multilevel analysis. *PLoS One* 2017;**12**:e0177091. doi:10.1371/journal.pone.0177091

42 Bokade CM, Madhura AD, Bagul AS, *et al.* Predictors of mortality in children due to severe and very severe pneumonia. *Niger Med J* 2015;**56**:287.

43 National Institute of Population Research and Training - NIPORT/Bangladesh, Mitra and Associates, ICF International. *Bangladesh Demographic and Health Survey 2014*. Dhaka,

For peer review only - http://bmjopen.bmj.com/site/about/guidelines.xhtml

Bangladesh: NIPORT, Mitra and Associates, and ICF International 2016. http://dhsprogram.com/pubs/pdf/FR311/FR311.pdf

44 Christakis DA, Mell L, Wright JA, *et al.* The association between greater continuity of care and timely measles-mumps-rubella vaccination. *Am J Public Health* 2000;**90**:962.

45 Christakis DA, Mell L, Koepsell TD, *et al.* Association of lower continuity of care with greater risk of emergency department use and hospitalization in children. *Pediatrics* 2001;**107**:524–9.

46 Van Walraven C, Oake N, Jennings A, *et al.* The association between continuity of care and outcomes: a systematic and critical review. *J Eval Clin Pract* 2010;**16**:947–56.

47 Kuhn R. Identities in motion: Social exchange networks and rural- urban migration in Bangladesh. *Contributions to Indian Sociology* 2003;**37**:311–37. doi:10.1177/006996670303700113

48 Mills E, Jadad AR, Ross C, *et al.* Systematic review of qualitative studies exploring parental beliefs and attitudes toward childhood vaccination identifies common barriers to vaccination. *J Clin Epidemiol* 11;**58**:1081–8. doi:10.1016/j.jclinepi.2005.09.002

49 Rainey JJ, Watkins M, Ryman TK, *et al.* Reasons related to non-vaccination and under-vaccination of children in low and middle income countries: findings from a systematic review of the published literature, 1999–2009. *Vaccine* 2011;**29**:8215–21.

50 Favin M, Steinglass R, Fields R, *et al.* Why children are not vaccinated: a review of the grey literature. *International Health* 2012;**4**:229–38. doi:10.1016/j.inhe.2012.07.004

51 Sharma V, Singh A, Sharma V. Provider's and User's Perspective about Immunization Coverage among Migratory and Non-migratory Population in Slums and Construction Sites of Chandigarh. *J Urban Health* 2015;**92**:304–12. doi:10.1007/s11524-015-9939-2

For peer review only

For peer review only - http://bmjopen.bmj.com/site/about/guidelines.xhtml

Figures and Tables

Table 1: **Mobility status of study households with children age 0-59 months and association with demographic, socioeconomic, and health characteristics using t- and χ^2-tests**

	Residentially stable, ≥24 months n=5513		Recently relocated, ≤12 months n=4507		p-value
Demographics	mean	SE	mean	SE	
Number of household members	5.40	0.09	4.60	0.10	**<0.001**
Number of children <5yrs in household	1.28	0.01	1.25	0.01	0.194
Age of index child in months	30.0	0.23	28.7	0.25	**<0.001**
	n	%	n	%	
Sex of index child: Male	2478	45	2048	45	0.622
Socioeconomics					
Mother's education					**<0.001**
No education	1133	21	1162	26	
Some schooling	1142	21	1176	26	
Finished secondary	1849	34	1483	33	
> Secondary	1389	25	686	15	
Father's education					**<0.001**
No education	1261	23	1139	25	
Some schooling	913	17	911	20	
Finished secondary	1525	28	1372	30	
> Secondary	1814	33	1085	24	
Occupation of household head					**<0.001**
Unemployed or other	482	9	232	5	
Daily labor	916	17	1218	27	
Shopkeeper or merchant	1787	32	1058	23	
Salaried service	2328	42	1999	44	
Monthly household income[a]					**<0.001**
≤ 5,000 taka (US $73)	971	18	1080	24	

For peer review only

For peer review only - http://bmjopen.bmj.com/site/about/guidelines.xhtml

5,001 – 10,000 taka	1634	30	1925	43	
> 10,000 taka (US $145)	2900	53	1498	33	
Household wealth index[b]					**<0.001**
Lowest	1092	20	1110	25	
Second	922	17	1021	23	
Third	966f	18	911	20	
Fourth	1455	26	1155	26	
Highest	1078	20	310	7	
Health services knowledge					
Knowledge of local hospital	4709	85	3428	76	**<0.001**
Health outcomes					
Severe acute respiratory illness suffered by index child within 12 months[c]	763	14	695	15	**0.026**
Meningitis/encephalitis suffered by index child within 12 months[d]	185	3	164	4	0.443

[a]12 respondents (8 residentially stable and 4 recently relocated) did not know or did not disclose household income.
[b]Polychoric Principal Components Analysis was used to create a household wealth index including structural housing characteristics, cooking fuel, drinking water, and sanitation.
[c]One residentially stable respondent did not know if child recently had a severe acute respiratory illness.
[d]Three respondents did not know if child recently had a serious illness with mental status changes (2 residentially stable and 1 recently relocated).

For peer review only - http://bmjopen.bmj.com/site/about/guidelines.xhtml

Table 2: ***Using vaccination card plus maternal recall*****, vaccination coverage among children age 9-59 months and association with mobility status using univariate and multivariable models with modified Poisson regression**

	Partial vaccination n=1465 (17%)		Full vaccination[a] n=7043 (83%)		PR[b]	95% CI	p-value
Univariate analyses							
Mobility status	n	%	n	%			
Residentially stable ≥24 months	706	15	4007	85	Reference		
Recently relocated ≤12 months	759	20	3036	80	**0.94**	**0.91-0.97**	**<0.001**
Demographics	mean	SE	mean	SE			
Number of children <5yrs in household	1.31	0.02	1.25	0.01	**0.96**	**0.93-0.98**	**0.002**
Age of index child in months	32.0	0.44	34.2	0.16	**1.002**	**1.001-1.003**	**<0.001**
Socioeconomics							
Mother's education	n	%	n	%			
No education	629	31	1377	69	Reference		
Some schooling	401	21	1540	79	**1.16**	**1.10-1.21**	**<0.001**
Finished secondary	323	11	2513	89	**1.29**	**1.23-1.36**	**<0.001**
> Higher secondary	112	6	1613	94	**1.36**	**1.30-1.43**	**<0.001**
Occupation of household head							
Unemployed or other	106	17	505	83	Reference		
Daily labor	524	29	1283	71	**0.86**	**0.82-0.90**	**<0.001**
Shopkeeper or merchant	348	14	2084	86	1.04	0.99-1.08	0.112
Salaried service	487	13	3171	87	**1.05**	**1.01-1.09**	**0.029**
Household wealth status (PCA[c])							
Lowest	596	32	1290	68	Reference		
Second	334	20	1298	80	**1.16**	**1.09-1.24**	**<0.001**
Third	242	15	1348	85	**1.24**	**1.16-1.32**	**<0.001**
Fourth	193	9	2043	91	**1.34**	**1.25-1.42**	**<0.001**
Highest	100	9	1064	91	**1.34**	**1.25-1.43**	**<0.001**
Health services knowledge							
Does *not* have knowledge of local hospital	354	23	1218	77	Reference		
Has knowledge of local hospital	1111	16	5825	84	**1.08**	**1.05-1.12**	**<0.001**

For peer review only - http://bmjopen.bmj.com/site/about/guidelines.xhtml

Multivariable analyses with different models				
Mobility, adjusting for demographics (# of children and age of index child)		**0.94**	**0.92-0.97**	**<0.001**
Mobility, adjusting for socioeconomics (education, occupation, and wealth)		**0.97**	**0.95-0.99**	**0.009**
Mobility, adjusting for demographics and socioeconomics		**0.97**	**0.95-0.99**	**0.016**

[a]Full vaccination coverage per Expanded Programme on Immunization includes 1 dose of BCG, 3 doses of polio, 3 doses of DPT, and 1 dose of measles vaccines.
[b]PR, Prevalence Ratio.
[c]Polychoric Principal Components Analysis was used to create a household wealth index including structural housing characteristics, cooking fuel, drinking water, and sanitation.

For peer review only

Table 3: *Using vaccination card only*, vaccination coverage among children age 9-59 months who have vaccination cards and association with mobility status using univariate and multivariable models with modified Poisson regression

	Partial vaccination n=564 (15%)		Full vaccination[a] n=3085 (85%)		PR[b]	95% CI	p-value
Univariate analyses							
Mobility status	n	%	n	%			
Residentially stable ≥24 months	329	14	1948	86	Reference		
Recently relocated ≤12 months	235	17	1137	83	0.97	0.93-1.00	0.083
Demographics	mean	SE	mean	SE			
Number of children <5yrs in household	1.26	0.02	1.24	0.01	0.99	0.96-1.02	0.486
Age of index child in months	27.1	0.64	29.6	0.25	**1.002**	**1.001-1.003**	**0.001**
Socioeconomics							
Mother's education	n	%	n	%			
No education	165	28	430	72	Reference		
Some schooling	123	16	654	84	**1.16**	**1.09-1.24**	**<0.001**
Finished secondary	186	14	1158	86	**1.19**	**1.11-1.28**	**<0.001**
> Higher secondary	90	10	843	90	**1.25**	**1.17-1.33**	**<0.001**
Occupation of household head							
Unemployed or other	35	13	238	87	Reference		
Daily labor	148	26	413	74	**0.84**	**0.78-0.91**	**<0.001**
Shopkeeper or merchant	143	13	921	87	0.99	0.94-1.04	0.778
Salaried service	238	14	1513	86	0.99	0.95-1.04	0.704
Household wealth status (PCA[c])							
Lowest	148	27	401	73	Reference		
Second	131	21	503	79	**1.09**	**1.02-1.16**	**0.009**
Third	97	15	572	86	**1.17**	**1.09-1.26**	**<0.001**
Fourth	110	10	1005	90	**1.23**	**1.15-1.32**	**<0.001**
Highest	78	11	604	89	**1.21**	**1.13-1.30**	**<0.001**
Health services knowledge							
Does *not* have knowledge of local hospital	101	19	434	81	Reference		
Has knowledge of local hospital	463	15	2651	85	**1.05**	**1.01-1.09**	**0.025**

For peer review only

For peer review only - http://bmjopen.bmj.com/site/about/guidelines.xhtml

Multivariable analyses with different models			
Mobility, adjusting for demographics (# of children and age of index child)	0.97	0.94-1.01	0.126
Mobility, adjusting for socioeconomics (education, occupation, and wealth)	0.98	0.95-1.02	0.308
Mobility, adjusting for demographics and socioeconomics	0.98	0.95-1.02	0.396

[a]Full vaccination coverage per Expanded Programme on Immunization includes 1 dose of BCG, 3 doses of polio, 3 doses of DPT, and 1 dose of measles vaccines.
[b]PR, Prevalence Ratio.
[c]Polychoric Principal Components Analysis was used to create a household wealth index including structural housing characteristics, cooking fuel, drinking water, and sanitation.

For peer review only

For peer review only - http://bmjopen.bmj.com/site/about/guidelines.xhtml

Table 4: Qualified provider visits for severe acute respiratory illness within prior year among children < 5 years old and association with mobility using univariate and multivariable models with modified Poisson regression

	No qualified provider for severe ARI[a] n=358 (25%)		Qualified provider for severe ARI[a] n=1100 (75%)		PR[b]	95% CI	p-value
Univariate analyses							
Mobility status	n	%	n	%			
Residentially stable ≥24 months	141	18	622	82	Reference		
Recently relocated ≤12 months	217	31	478	69	**0.84**	**0.79-0.90**	**<0.001**
Demographics	mean	SE	mean	SE			
Number of children <5yrs in household	1.23	0.03	1.25	0.02	1.02	0.97-1.08	0.437
Age of index child in months	29.1	0.83	23.7	0.44	**0.994**	**0.992-0.996**	**<0.001**
Socioeconomics							
Mother's education	n	%	n	%			
No education	134	38	220	62	Reference		
Some schooling	127	32	267	68	1.09	0.96-1.23	0.170
Finished secondary	79	16	425	84	**1.36**	**1.20-1.54**	**<0.001**
> Higher secondary	18	9	188	91	**1.47**	**1.30-1.66**	**<0.001**
Occupation of household head							
Unemployed or other	24	23	82	77	Reference		
Daily labor	139	36	244	64	**0.82**	**0.72-0.94**	**0.005**
Shopkeeper or merchant	89	22	320	78	1.01	0.90-1.14	0.848
Salaried service	106	19	454	81	1.05	0.93-1.18	0.435
Household wealth status (PCA[c])							
Lowest	154	40	228	60	Reference		
Second	93	30	220	70	**1.18**	**1.02-1.37**	**0.031**
Third	59	21	220	79	**1.32**	**1.16-1.51**	**<0.001**
Fourth	37	12	277	88	**1.48**	**1.31-1.67**	**<0.001**
Highest	15	9	155	91	**1.53**	**1.35-1.73**	**<0.001**
Health services knowledge							
Does *not* have knowledge of local hospital	114	49	118	51	Reference		

For peer review only - http://bmjopen.bmj.com/site/about/guidelines.xhtml

For peer review only

Has knowledge of local hospital	244	20	982	80	**1.57**	**1.34-1.85**	**<0.001**
Multivariable analyses with different models							
Mobility, adjusting for demographics (# of children and age of index child)					**0.84**	**0.79-0.89**	**<0.001**
Mobility, adjusting for socioeconomics (education, occupation, and wealth)					**0.89**	**0.84-0.94**	**<0.001**
Mobility, adjusting for demographics and socioeconomics					**0.88**	**0.84-0.93**	**<0.001**

[a]ARI, Acute Respiratory Illness.
[b]PR, Prevalence Ratio.
[c]Polychoric Principal Components Analysis was used to create a household wealth index including structural housing characteristics, cooking fuel, drinking water, and sanitation.

For peer review only

For peer review only - http://bmjopen.bmj.com/site/about/guidelines.xhtml

Figure 1: Children sampled by mobility status for healthcare utilization and vaccination coverage analysis
Note: *ARI = Acute Respiratory Illness

For peer review only

For peer review only - http://bmjopen.bmj.com/site/about/guidelines.xhtml

Supplemental Figure 1: Cook's distance calculations to examine influential observations

Cook's distance plots of multivariable models examining mobility, demographics, and socioeconomics for: vaccination using card plus maternal recall among children 9-59 months (A), vaccination using card only among children 9-59 months (B), qualified provider visits for respiratory infections among children <5 years old (C), vaccination using card plus maternal recall among children 9-23 months (D), and vaccination using card only among children 9-23 months (E). The red lines indicate threshold value of 4/n where n=number of observations.

For peer review only

For peer review only - http://bmjopen.bmj.com/site/about/guidelines.xhtml

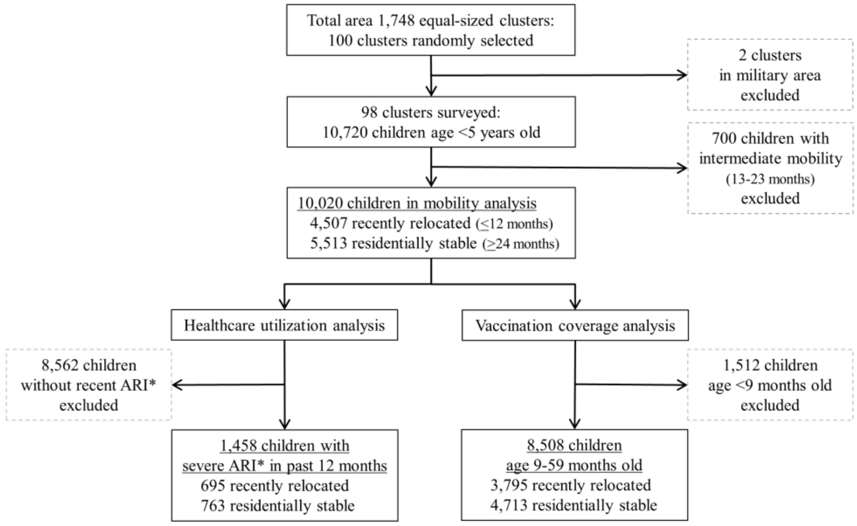

Figure 1: Children sampled by mobility status for healthcare utilization and vaccination coverage analysis
Note: *ARI = Acute Respiratory Illness

Figure 1: Children sampled by mobility status for healthcare utilization and vaccination coverage analysis
Note: *ARI = Acute Respiratory Illness

254x190mm (300 x 300 DPI)

Effect of household relocation on child vaccination and health service utilization in Dhaka, Bangladesh: a cross-sectional community survey

SUPPLEMENTAL FIGURES AND TABLES

Supplemental Table 1: Household wealth principal components analysis coding and variable loading

Indicator	Coding	Loading
Number of rooms	Continuous	0.337
Housing arrangement	1 = Free<br>2 = Rental<br>3 = Owned	0.235
Roof	1 = Natural roof (bamboo/thatch)<br>2 = Rudimentary roof (tin)<br>3 = Finished roof (cement/concrete/tiled)<br>4 = Other	0.392
Walls	1 = Natural walls (jute/bamboo/mud)<br>2 = Rudimentary walls (wood)<br>3 = Finished walls (tin/brick/cement)<br>4 = Other	0.316
Floor	1 = Natural floor (earth/bamboo)<br>2 = Rudimentary floor (wood)<br>3 = Finished floor (cement/concrete)<br>4 = Other	0.377
Cooking fuel	1 = Natural (wood/grass/dung)<br>2 = Coal/charcoal<br>3 = Kerosene<br>4 = Electricity<br>5 = Gas (liquid/biogas)<br>6 = Other	0.350
Drinking water	1 = Unimproved (unprotected/surface/bottled)<br>2 = Improved but not piped<br>3 = Improved and piped into yard or private dwelling<br>4 = Other	0.170
Sanitation	1 = Unimproved (open defecation/hanging/open or broken pit)<br>2 = Improved but not piped	0.374

For peer review only

For peer review only - http://bmjopen.bmj.com/site/about/guidelines.xhtml

	3 = Improved and piped sewer<br>4 = Other	
Mobile phone ownership	0 = No<br>1 = Yes	0.380

Note: 51% of overall variance was explained by the first component.

For peer review only

For peer review only - http://bmjopen.bmj.com/site/about/guidelines.xhtml

Supplemental Table 2: *Using vaccination card plus maternal recall*, vaccination coverage among children age 9-23 months and association with mobility status using univariate and multivariable models with modified Poisson regression

	Partial vaccination n=518 (21%)		Full vaccination[a] n=1906 (79%)		PR[b]	95% CI	p-value
Univariate analyses							
Mobility status	n	%	n	%			
Residentially stable ≥24 months	243	19	1053	81	Reference		
Recently relocated ≤12 months	275	24	853	76	**0.93**	**0.88-0.98**	**0.009**
Demographics	mean	SE	mean	SE			
Number of children <5yrs in household	1.31	0.03	1.24	0.01	**0.94**	**0.89-0.99**	**0.035**
Age of index child in months	14.73	0.20	16.35	0.11	**1.02**	**1.02-1.03**	**<0.001**
Socioeconomics							
Mother's education	n	%	n	%			
No education	207	41	303	59	Reference		
Some schooling	121	22	433	78	**1.32**	**1.18-1.47**	**<0.001**
Finished secondary	141	16	719	84	**1.41**	**1.27-1.56**	**<0.001**
> Higher secondary	49	10	451	90	**1.52**	**1.36-1.69**	**<0.001**
Occupation of household head							
Unemployed or other	26	15	147	85	Reference		
Daily labor	195	38	318	62	**0.73**	**0.66-0.81**	**<0.001**
Shopkeeper or merchant	124	19	538	81	0.96	0.88-1.04	0.287
Salaried service	173	16	903	84	0.99	0.91-1.07	0.752
Household wealth status (PCA[c])							
Lowest	212	40	321	60	Reference		
Second	114	25	348	75	**1.25**	**1.11-1.40**	**<0.001**
Third	84	18	382	82	**1.36**	**1.20-1.54**	**<0.001**
Fourth	59	10	560	90	**1.50**	**1.34-1.69**	**<0.001**
Highest	49	14	295	86	**1.42**	**1.26-1.61**	**<0.001**
Health services knowledge							
Does *not* have knowledge of local hospital	127	28	324	72	Reference		
Has knowledge of local hospital	391	20	1582	80	**1.12**	**1.05-1.19**	**<0.001**

For peer review only

For peer review only - http://bmjopen.bmj.com/site/about/guidelines.xhtml

Multivariable analyses with different models				
Mobility, adjusting for demographics (# of children and age of index child)		**0.93**	**0.88-0.98**	**0.007**
Mobility, adjusting for socioeconomics (education, occupation, and wealth)		0.96	0.91-1.01	0.136
Mobility, adjusting for demographics and socioeconomics		0.96	0.92-1.01	0.138

[a]Full vaccination coverage per Expanded Programme on Immunization includes 1 dose of BCG, 3 doses of polio, 3 doses of DPT, and 1 dose of measles vaccines.
[b]PR, Prevalence Ratio.
[c]Polychoric Principal Components Analysis was used to create a household wealth index including structural housing characteristics, cooking fuel, drinking water, and sanitation.

For peer review only

Supplemental Table 3: *Using vaccination card only*, vaccination coverage among children age 9-23 months who have vaccination cards and association with mobility status using univariate and multivariable models with modified Poisson regression

	Partial vaccination n=281 (19%)		Full vaccination[a] n=1222 (81%)		PR[b]	95% CI	p-value
Univariate analyses							
Mobility status	n	%	n	%			
Residentially stable ≥24 months	156	18	715	82	Reference		
Recently relocated ≤12 months	125	20	507	80	0.97	0.92-1.04	0.469
Demographics	mean	SE	mean	SE			
Number of children <5yrs in household	1.23	0.03	1.21	0.01	0.98	0.93-1.04	0.543
Age of index child in months	14.4	0.27	15.9	0.15	**1.02**	**1.01-1.03**	**<0.001**
Socioeconomics							
Mother's education	n	%	n	%			
No education	94	35	176	65	Reference		
Some schooling	57	17	286	83	**1.28**	**1.15-1.42**	**<0.001**
Finished secondary	93	17	461	83	**1.28**	**1.14-1.43**	**<0.001**
> Higher secondary	37	11	299	89	**1.37**	**1.22-1.52**	**<0.001**
Occupation of household head							
Unemployed or other	14	12	100	88	Reference		
Daily labor	89	33	184	67	**0.77**	**0.68-0.87**	**<0.001**
Shopkeeper or merchant	66	16	352	84	0.96	0.88-1.04	0.327
Salaried service	112	16	586	84	0.96	0.88-1.04	0.297
Household wealth status (PCA[c])							
Lowest	84	32	179	68	Reference		
Second	67	24	211	76	1.12	0.99-1.25	0.062
Third	52	17	248	83	**1.21**	**1.08-1.37**	**0.002**
Fourth	42	10	381	90	**1.32**	**1.18-1.48**	**<0.001**
Highest	36	15	203	85	**1.25**	**1.10-1.41**	**<0.001**
Health services knowledge							
Does *not* have knowledge of local hospital	55	22	194	78	Reference		
Has knowledge of local hospital	226	18	1028	82	1.05	0.98-1.13	0.141

For peer review only - http://bmjopen.bmj.com/site/about/guidelines.xhtml

Multivariable analyses with different models				
Mobility, adjusting for demographics (# of children and age of index child)		0.98	0.93-1.04	0.582
Mobility, adjusting for socioeconomics (education, occupation, and wealth)		1.00	0.94-1.06	0.948
Mobility, adjusting for demographics and socioeconomics		1.00	0.95-1.07	0.888

[a]Full vaccination coverage per Expanded Programme on Immunization includes 1 dose of BCG, 3 doses of polio, 3 doses of DPT, and 1 dose of measles vaccines.
[b]PR, Prevalence Ratio.
[c]Polychoric Principal Components Analysis was used to create a household wealth index including structural housing characteristics, cooking fuel, drinking water, and sanitation.

For peer review only

For peer review only - http://bmjopen.bmj.com/site/about/guidelines.xhtml

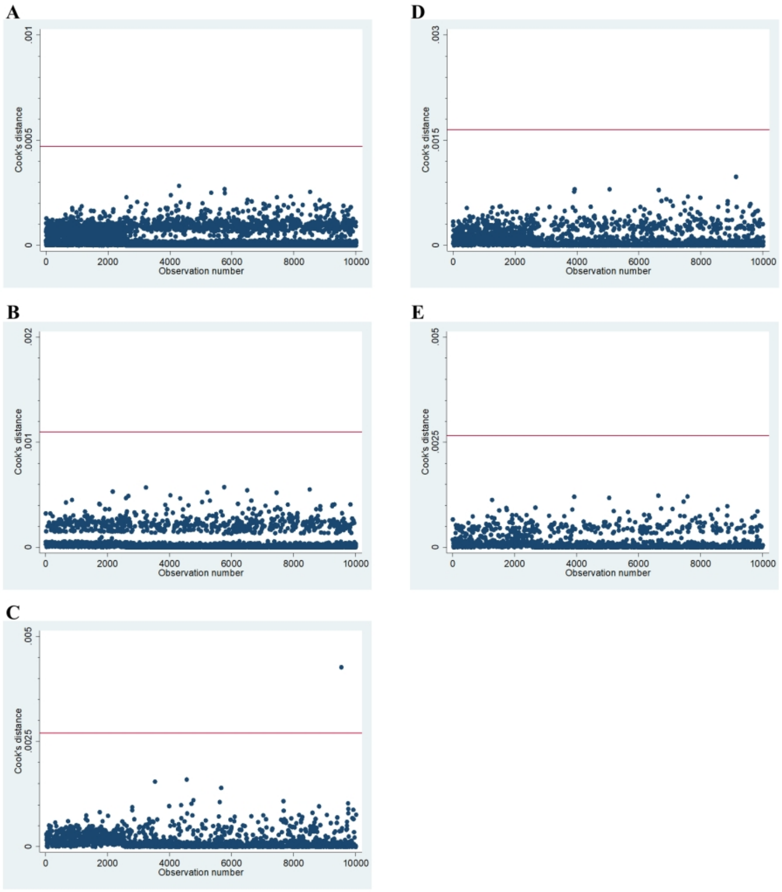

Supplemental Figure 1: Cook's distance calculations to examine influential observations
Cook's distance plots of multivariable models examining mobility, demographics, and socioeconomics for: vaccination using card plus maternal recall among children 9-59 months (A), vaccination using card only among children 9-59 months (B), qualified provider visits for respiratory infections among children <5 years old (C), vaccination using card plus maternal recall among children 9-23 months (D), and vaccination using card only among children 9-23 months (E). The red lines indicate threshold value of 4/n where n=number of observations.

190x254mm (300 x 300 DPI)

Effect of household relocation on child vaccination and health service utilization in Dhaka, Bangladesh: a cross-sectional community survey

STROBE 2007 (v4) Statement—Checklist of items that should be included in reports of *cross-sectional studies*

Section/Topic	Item #	Recommendation	Reported on page #
**Title and abstract**	1	(*a*) Indicate the study's design with a commonly used term in the title or the abstract	1
		(*b*) Provide in the abstract an informative and balanced summary of what was done and what was found	2
**Introduction**			
Background/rationale	2	Explain the scientific background and rationale for the investigation being reported	4
Objectives	3	State specific objectives, including any prespecified hypotheses	6
**Methods**			
Study design	4	Present key elements of study design early in the paper	6
Setting	5	Describe the setting, locations, and relevant dates, including periods of recruitment, exposure, follow-up, and data collection	6
Participants	6	(*a*) Give the eligibility criteria, and the sources and methods of selection of participants	7
Variables	7	Clearly define all outcomes, exposures, predictors, potential confounders, and effect modifiers. Give diagnostic criteria, if applicable	7
Data sources/ measurement	8*	For each variable of interest, give sources of data and details of methods of assessment (measurement). Describe comparability of assessment methods if there is more than one group	7
Bias	9	Describe any efforts to address potential sources of bias	8
Study size	10	Explain how the study size was arrived at	7
Quantitative variables	11	Explain how quantitative variables were handled in the analyses. If applicable, describe which groupings were chosen and why	8
Statistical methods	12	(*a*) Describe all statistical methods, including those used to control for confounding	8
		(*b*) Describe any methods used to examine subgroups and interactions	8
		(*c*) Explain how missing data were addressed	8
		(*d*) If applicable, describe analytical methods taking account of sampling strategy	8

For peer review only

		(*e*) Describe any sensitivity analyses	8
**Results**			
Participants	13*	(a) Report numbers of individuals at each stage of study—eg numbers potentially eligible, examined for eligibility, confirmed eligible, included in the study, completing follow-up, and analysed	9
		(b) Give reasons for non-participation at each stage	9
		(c) Consider use of a flow diagram	Figure 1
Descriptive data	14*	(a) Give characteristics of study participants (eg demographic, clinical, social) and information on exposures and potential confounders	9
		(b) Indicate number of participants with missing data for each variable of interest	9
Outcome data	15*	Report numbers of outcome events or summary measures	9
Main results	16	(*a*) Give unadjusted estimates and, if applicable, confounder-adjusted estimates and their precision (eg, 95% confidence interval). Make clear which confounders were adjusted for and why they were included	10
		(*b*) Report category boundaries when continuous variables were categorized	9
		(*c*) If relevant, consider translating estimates of relative risk into absolute risk for a meaningful time period	n/a
Other analyses	17	Report other analyses done—eg analyses of subgroups and interactions, and sensitivity analyses	10
**Discussion**			
Key results	18	Summarise key results with reference to study objectives	11
Limitations	19	Discuss limitations of the study, taking into account sources of potential bias or imprecision. Discuss both direction and magnitude of any potential bias	13
Interpretation	20	Give a cautious overall interpretation of results considering objectives, limitations, multiplicity of analyses, results from similar studies, and other relevant evidence	15
Generalisability	21	Discuss the generalisability (external validity) of the study results	14
**Other information**			
Funding	22	Give the source of funding and the role of the funders for the present study and, if applicable, for the original study on which the present article is based	17

*Give information separately for cases and controls in case-control studies and, if applicable, for exposed and unexposed groups in cohort and cross-sectional studies.

Note: An Explanation and Elaboration article discusses each checklist item and gives methodological background and published examples of transparent reporting. The STROBE checklist is best used in conjunction with this article (freely available on the Web sites of PLoS Medicine at http://www.plosmedicine.org/, Annals of Internal Medicine at http://www.annals.org/, and Epidemiology at http://www.epidem.com/). Information on the STROBE Initiative is available at www.strobe-statement.org.

For peer review only
